# Supplementary material for: Demographic and socioeconomic patterns in healthcare-seeking behaviour for respiratory symptoms in England: a comparison with non-respiratory symptoms and between three healthcare services
Source: BMJ Open. 2020 Nov 6;10(11):e038356. doi: 10.1136/bmjopen-2020-038356 (PMC7651740; doi:10.1136/bmjopen-2020-038356)
Supplement: Supplementary data [file bmjopen-2020-038356supp001.pdf]

BMJ Publishing Group Limited (BMJ) disclaims all liability and responsibility arising from any reliance placed on this supplemental material which has been supplied by the author(s)

Supplementary Material

Appendix 1. Descriptions of Syndromic Indicators used in study

Healthcare Service	Syndromic Indicator	Description
NHS111	Difficulty breathing	Patients calling NHS111 and reporting symptoms of breathing problems, breathlessness or wheeze<br>This syndromic indicator excludes those callers reporting symptoms of difficulty breathing which the call handler assesses as being of immediate threat to life for which an emergency ambulance is required. These patients would not routinely continue with telephone triage and therefore would not have a Pathway selected.
	Cough	Patients calling NHS111 and reporting symptoms of cough
	Cold/flu	Patients calling NHS111 and reporting symptoms of colds and/or influenza
GPIH	Upper respiratory tract infection (URTI)	Codes suggestive of an acute bacterial or viral infection mainly affecting the upper respiratory tract (includes colds/flu, sinusitis, throat infections, ear infections).
	Lower respiratory tract infection (LRTI)	Codes suggestive of an acute bacterial or viral infection mainly affecting the lower respiratory tract including pneumonia, bronchitis, bronchiolitis, pleurisy and complications. Includes codes for the pneumonia indicator.
GPOOH	Asthma	Diagnoses of acutely presenting or severe asthma including asthma attack, not including routine consultations (where possible to distinguish and not including prescription links)
	Acute respiratory infection (ARI)	Acute respiratory infection - includes all codes indicative of an acute respiratory infection eg. acute sinusitis, viral pneumonia, influenza and pleurisy
	Difficulty breathing/wheeze/asthma	Difficulty breathing/wheeze/asthma - includes codes indicative of asthma, wheeze and difficulty breathing. Includes eg. dyspnoea, bronchial breathing, expiratory wheeze and stridor.

Appendix 2. Spatial variation of contacts to three health services in England between 1st January 2015 & 31st December 2016

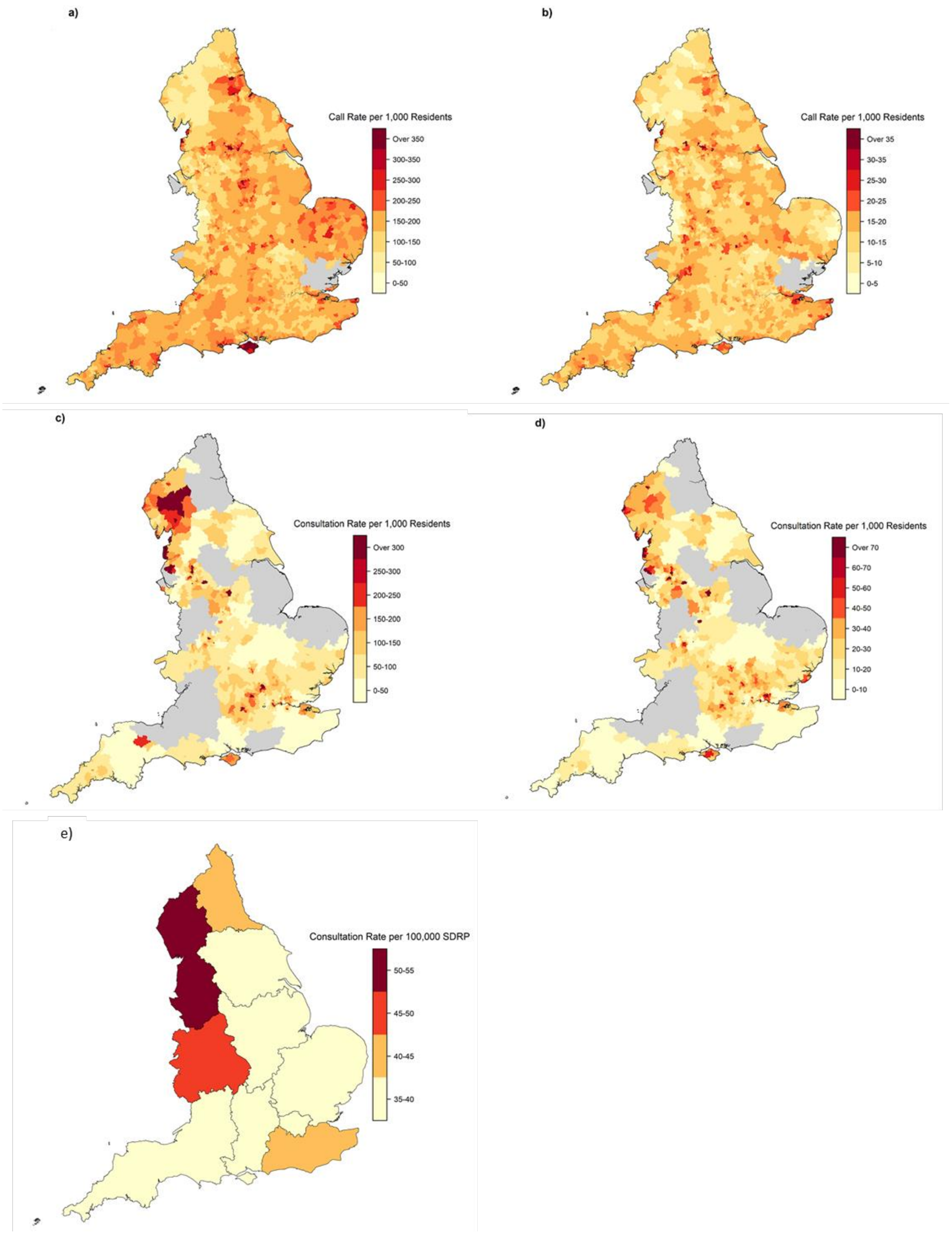

a) All non-respiratory calls to NHS111 at PD,
b) Respiratory calls to NHS111 at PD,
c) All non-respiratory consultations to GPOOH services at PD,
d) Respiratory consultations to GPOOH services at PD,
e) Respiratory calls to GPIH services PHE centre. SDPR = Sum of daily registered population
The grey areas indicate the PD that were excluded for the duration of the study period.

Appendix 3. Comparison between GLM and GLMM results of respiratory calls to GPIH (ns = not significant (overall effect only), * = ≤0.05, ** = ≤0.01, *** = ≤0.001).

	GLM		GLMM	
Variable	**Rate** Ratio	95% CI	**Rate** Ratio	95% CI
Intercept	142.693	0.000 –1.455e+16	142.264	0.000-2.619e+16
Main Effects				
Age		***		***
Under 1 year	6.213	5.955 – 6.482***	6.213	5.950-6.487***
1-4 years	3.492	3.348 – 3.643***	3.492	3.450-3.645***
5-14 years	1.172	1.123 – 1.223***	1.172	1.123 - 1.223***
15-44 years	ref	ref	ref	ref
45-64 years	1.032	0.989 – 1.076	1.032	0.988 - 1.077
65-74 years	1.170	1.122 – 1.221***	1.170	1.121 - 1.222***
75 years and above	1.500	1.438 – 1.564***	1.500	1.436 - 1.566***
Gender				***
Female	ref	ref	ref	ref
Male	0.513	0.492 – 0.535***	0.513	0.492-0.536***
PHE Centre	-	***	-	-
Year	-	ns	-	ns
Interaction Effects				
Age:Gender	-	***	-	***
AIC	5083.492		5117.537	
Deviance Explained	0.993		0.992	
Dispersion Statistic	1.100		1.0332	

Appendix 4. Map of upper teir local authorties over-layed postcode dictricts

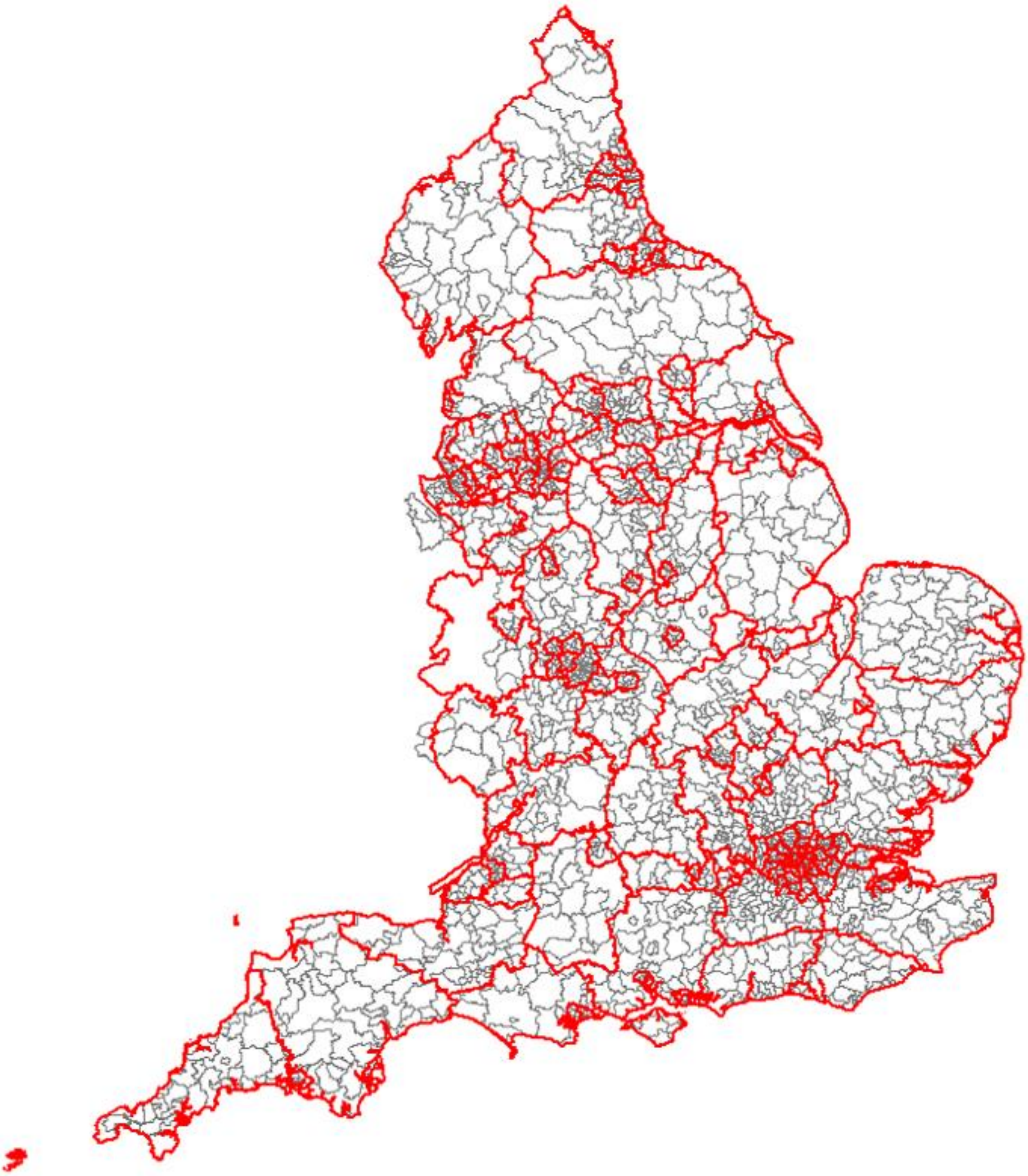

Upper-tier local authorities (red) overlapped over PD (grey)

Appendix 5. Algebraic definition of the models

The general algebraic definition of the models is given by:

$$Y_{a,g,i,y} \mid \mu_{a,g,i,y},\varphi \sim NegBin(\mu_{a,g,i,y},\varphi),$$

Where $\mu_{a,g,i,y}$ is the is the number of age a and gender g specific contacts at each geographical location (PHE centre or postcode district) i at year y, and $\varphi > 0$ is the negative binomial dispersion parameter. The expected number of cases for GPIH services is modelled as:

$$\log(\mu_{a,g,i,y}) = \alpha + \log(P_{a,g,i,y}) + \beta a + \gamma_g + \delta_y + \epsilon_i + \beta a \cdot \gamma_g$$

Where α corresponds to the intercept; $\log(P_{a,g,i,y})$ denotes the logarithm of the population P at risk for PHE centre i, age a, gender g and year y included as an offset to adjust counts by population; a denotes a categorical variable age with coefficient β; g denotes a categorical variable gender with coefficient γ; y denotes a categorical variable for each year with coefficient δ; i denotes a categorical variable PHE centre with coefficient ϵ. The interaction between age and gender is denoted by $\beta a \cdot \gamma_g$.

The expected number of cases for NHS111 and GPOOH services is modelled as:

$$\log(\mu a,g,i,y) = \alpha + \log(Pa,g,i,y) + \beta a + \gamma g + \delta y + \zeta d + \eta p + \beta a \cdot \gamma g + \beta a \cdot \zeta d + ui + vl$$

Where α corresponds to the intercept; $\log(P_{a,g,i,y})$ denotes the logarithm of the population P at risk for postcode district i, age a, gender g and year y included as an offset to adjust counts by population at risk; a denotes a categorical variable age with coefficient β; g denotes a categorical variable gender with coefficient γ; y denotes a categorical variable for year with coefficient δ; d denotes a categorical variable for deprivation with coefficient ζ; p denotes a continuous variable to represent the percentage of postcode district that is classified as urban with coefficient η. $\beta a \cdot \gamma_g$ denotes the interaction between age and gender, and $\beta a \cdot \zeta_d$ denotes the interaction between age and deprivation. Unstructured random effects of postcode district (u_i) and upper tier local authority (v_l) were included to account for unknown confounding factors, such as temperature, the hierarchical structure of the data and spatial dependency.

Appendix 6. Cross validation of final model to assess model overfitting.

	NSH111		GPOOH		GPIH
	Respiratory	Non-Respiratory	Respiratory	Non-Respiratory	Respiratory
Max number of contacts per location	391	17,842	2,108	10,175	200,304
Min number of contacts per location	0	0	0	0	4,274
Mean number of contacts per location	30.9	350.3	39.2	173.9	36,823.7
Mean of the mean absolute error	7.02	54.6	9.5	40.5	2,533.7

Appendix 7. Final Model multivariable regression analysis of respiratory and non-respiratory contacts to three healthcare services. Full results and overall significance presented for main effects, and overall significance for interaction terms (ns = not significant, * = ≤0.05, ** = ≤0.01, *** = ≤0.001, x = variable not modelled, - = reference level).

	NHS 111				GPOOH				GPIH	
	Respiratory Calls		Non-respiratory Calls		Respiratory Calls		Non-respiratory Calls		Respiratory Calls (PHE Centre	
**Variable**	**Rate Ratio**	**95% CI**	**Rate Ratio**	**95% CI**	**Rate Ratio**	**95% CI**	**Rate Ratio**	**95% CI**	**Rate Ratio**	**95% CI**
Intercept	0.005	0.004-0.005	0.106	0.099-0.115	0.002	0.001-0.003***	0.015	0.009-0.023***	142.693	0.000 – 1.455e+16
**Main Effects**										
%Urban	1.002	1.002-1.003***	1.001	1.001-1.002***	1.004	1.003-1.006***	1.004	1.003-1.006***	x	x
Age	–	***	–	***	–	***	–	***	–	***
Under 1 year	37.324	36.104-38.850***	7.031	6.869-7.197***	18.661	17.783-19.581***	7.640	7.281-8.018***	6.213	5.955 – 6.482***
1 – 4 years	16.683	16.177-17.206***	3.220	3.149-3.292***	9.822	9.400-10.263***	2.782	2.656-2.913***	3.492	3.348 – 3.643***
5 – 14 years	1.983	1.915-2.054***	0.871	0.851-0.891***	1.879	1.794-1.966***	0.791	0.755-0.829***	1.172	1.123 – 1.223***
15-44 years	ref	ref	ref	ref	ref	ref	ref	ref	ref	ref
45-64 years	0.854	0.826-0.883***	0.670	0.656-0.685***	0.515	0.492-0.540***	0.795	0.761-0.831***	1.032	0.989 – 1.076
65-74 years	1.372	1.324-1.423***	0.912	0.892-0.932***	0.674	0.640-0.709***	1.247	1.192-1.304***	1.170	1.122 – 1.221***
75 years and over	3.999	3.872-4.130***	2.673	2.616-2.732***	2.500	2.388-2.617***	4.029	3.855-4.211***	1.500	1.438 – 1.564***
Deprivation Quintile	–	***	–	***	–	***	–	***	–	–
(Most Deprived) 1	1.745	1.561-1.949***	1.814	1.658-1.985***	2.704	1.792-4.081***	2.700	1.892-3.853***	x	x
2	1.382	1.291-1.480***	1.387	1.313-1.466***	1.654	1.326-2.062***	1.659	1.374-2.003***	x	x
3	1.172	1.104-1.245***	1.179	1.124-1.236***	1.318	1.089-1.596**	1.231	1.045-1.451*	x	x

4	1.075	1.019-1.133**	1.085	1.040-1.132***	1.153	0.986-1.153	1.200	1.050-1.372**	x	x
(Least Deprived) 5	ref	ref	ref	ref	ref	ref	ref	ref	x	x
Gender	–	***		***	–	***	–	***	–	–
Male	ref	ref	ref	ref	ref	ref	ref	ref	ref	ref
Female	1.588	1.558-1.618***	1.748	1.722-1.774***	1.733	1.697-1.770***	1.711	1.665-1.758	1.949	1.868-2.033***
PHE Centre	–	***	–	***	–	***	–	***	–	***
Year	–	***	–	***	–	***	–	***	–	ns
**Interaction Effects**										
Age:Gender	–	***	–	**	–	***	–	***	–	***
Age:Deprivation	–	***	–	***	–	***	–	***	x	x
**R-Squared**										
**Marginal**	0.864		0.813		0.303		0.227		0.993	
**Conditional**	0.941		0.915		0.959		0.945		-	
**Dispersion statistics**	1.1572		1.262		1.088		1.025		1.100	
