# Supplementary material for: Demographic and socioeconomic patterns in healthcare-seeking behaviour for respiratory symptoms in England: a comparison with non-respiratory symptoms and between three healthcare services
Source: BMJ Open. 2020 Nov 6;10(11):e038356. doi: 10.1136/bmjopen-2020-038356 (PMC7651740; doi:10.1136/bmjopen-2020-038356)
Supplement: Author's manuscript [file bmjopen-2020-038356.draft_revisions.pdf]

BMJ Open is committed to open peer review. As part of this commitment we make the peer review history of every article we publish publicly available.

When an article is published we post the peer reviewers' comments and the authors' responses online. We also post the versions of the paper that were used during peer review. These are the versions that the peer review comments apply to.

The versions of the paper that follow are the versions that were submitted during the peer review process. They are not the versions of record or the final published versions. They should not be cited or distributed as the published version of this manuscript.

BMJ Open is an open access journal and the full, final, typeset and author-corrected version of record of the manuscript is available on our site with no access controls, subscription charges or pay-per-view fees (http://bmjopen.bmj.com).

If you have any questions on BMJ Open's open peer review process please email info.bmjopen@bmj.com

BMJ Open

# Demographic and socioeconomic patterns in healthcare-seeking behaviour for respiratory symptoms in England; A comparison with non-respiratory symptoms and between three healthcare services

| | |
|---|---|
| Journal: | *BMJ Open* |
| Manuscript ID | bmjopen-2020-038356 |
| Article Type: | Original research |
| Date Submitted by the Author: | 07-Mar-2020 |
| Complete List of Authors: | Morrison, Kirsty; University of East Anglia, School of Environmental Sciences<br>Colón-González, Felipe ; University of East Anglia, School of Environmental Sciences<br>Morbey, Roger; Public Health England, Real-time Syndromic Surveillance Team<br>Hunter, Paul; University of East Anglia, Norwich Medical School<br>Rutter, Judith; NHS Digital<br>Stuttard, Gareth; NHS England and NHS Improvement<br>de Lusignan, Simon; University of Oxford, Nuffield Department of Primary Care Health Sciences; University of Surrey, Department of Clinical and Experimental Medicine<br>Yeates, Alex; Advanced<br>Pebody, Richard; Public Health England, Influenza and Other Respiratory Virus Section, Immunisation and Countermeasures Division, National Infection Service<br>Smith, Gillian; Public Health England, Real-time Syndromic Surveillance Team<br>Elliot, Alex; Public Health England, Real-time Syndromic Surveillance Team<br>Lake, Iain; University of East Anglia, School of Environmental Sciences |
| Keywords: | Epidemiology < INFECTIOUS DISEASES, STATISTICS & RESEARCH METHODS, Respiratory infections < THORACIC MEDICINE |
| | |

SCHOLARONE™
Manuscripts

*I, the Submitting Author has the right to grant and does grant on behalf of all authors of the Work (as defined in the below author licence), an exclusive licence and/or a non-exclusive licence for contributions from authors who are: i) UK Crown employees; ii) where BMJ has agreed a CC-BY licence shall apply, and/or iii) in accordance with the terms applicable for US Federal Government officers or employees acting as part of their official duties; on a worldwide, perpetual, irrevocable, royalty-free basis to BMJ Publishing Group Ltd ("BMJ") its licensees and where the relevant Journal is co-owned by BMJ to the co-owners of the Journal, to publish the Work in this journal and any other BMJ products and to exploit all rights, as set out in our licence.*

*The Submitting Author accepts and understands that any supply made under these terms is made by BMJ to the Submitting Author unless you are acting as an employee on behalf of your employer or a postgraduate student of an affiliated institution which is paying any applicable article publishing charge ("APC") for Open Access articles. Where the Submitting Author wishes to make the Work available on an Open Access basis (and intends to pay the relevant APC), the terms of reuse of such Open Access shall be governed by a Creative Commons licence – details of these licences and which Creative Commons licence will apply to this Work are set out in our licence referred to above.*

*Other than as permitted in any relevant BMJ Author's Self Archiving Policies, I confirm this Work has not been accepted for publication elsewhere, is not being considered for publication elsewhere and does not duplicate material already published. I confirm all authors consent to publication of this Work and authorise the granting of this licence.*

For peer review only

**Title:** Demographic and socioeconomic patterns in healthcare-seeking behaviour for respiratory symptoms in England; A comparison with non-respiratory symptoms and between three healthcare services

**Authors:**

Kirsty E Morrison[1], Felipe J Colón-González[1], Roger A Morbey[3], Paul R Hunter[2], Judith Rutter[4], Gareth Stuttard[5], Simon de Lusignan[6], Alex Yeates[7], Richard Pebody[8], Gillian Smith[3], Alex J. Elliot[3], Iain R Lake[1]

**Author Affiliations**

[1]School of Environmental Sciences, University of East Anglia, Norwich, Norfolk, United Kingdom.
[2]Norwich Medical School, University of East Anglia, Norwich, Norfolk, United Kingdom.
[3] Real-time Syndromic Surveillance Team, Field Service, National Infection Service, Public Health England, Birmingham, United Kingdom.
[4] NHS Digital, Leeds, United Kingdom.
[5] NHS England and NHS Improvement, London, United Kingdom.
[6] Nuffield Department of Primary Care Health Sciences, University of Oxford, Oxford, United Kingdom.
[7] Advanced, Ashford, United Kingdom.
[8] Influenza and Other Respiratory Virus Section, Immunisation and Countermeasures Division, National Infection Service, Public Health England, London, United Kingdom.

**Corresponding Author:**

Kirsty E Morrison,

School of Environmental Sciences,

University of East Anglia,

Norwich Research Park,

NR4 7TJ.

07850752299

k.morrison@uea.ac.uk

Word count: 2,998

**Key Words: Epidemiology; Statistics & Research Methods; Deprivation; Respiratory Infections**



For peer review only - http://bmjopen.bmj.com/site/about/guidelines.xhtml

Author Contributions:

Kirsty E Morrison – Responsible for conception and design of study, analysis and interpretation of data and drafting the manuscript.

Felipe J Colón-González – Significant input in the conception and design of study, analysis and interpretation of data and drafting the manuscript and critically reviewed the final version of the work

Roger A Morbey - Significant input in the conception and design of study, interpretation of data, drafting the manuscript and critically reviewed the final version of the work

Paul R Hunter - Critically reviewed the final version of the work

Judith Rutter - Critically reviewed the final version of the work

Gareth Stuttard - Critically reviewed the final version of the work

Simon de Lusignan - Critically reviewed the final version of the work

Alex Yeates - Critically reviewed the final version of the work

Richard Pebody - Critically reviewed the final version of the work

Gillian Smith - Significant input in the conception and design of study, interpretation of data, drafting the manuscript and critically reviewed the final version of the work

Alex J. Elliot - Significant input in the conception and design of study, interpretation of data, drafting the manuscript and critically reviewed the final version of the work

Iain R Lake - Significant input in the conception and design of study, analysis and interpretation of data and drafting the manuscript and critically reviewed the final version of the work

All authors approved the final version of the manuscript.

For peer review only



For peer review only - http://bmjopen.bmj.com/site/about/guidelines.xhtml

**Abstract**

*Objectives:* Respiratory disease is a global issue with over 2.3 million deaths in 2016 due to lower respiratory tract infections. This study will analyse respiratory related health care contacts to three community healthcare services that capture more of the disease burden than acute source of data such as hospital admissions. This study will explore associations between contact rates to these services and the patient's age, gender, and deprivation. Results will be compared between healthcare services, and with non-respiratory contacts to fully explore how these contacts differ by service and illness. It is crucial to investigate the social patterns in healthcare-seeking behaviour, enabling targeted public health interventions.

*Design*: Ecological Study

*Setting*: Respiratory contacts to three healthcare services in England (NHS111; a telehealth helpline, and GPIH; family doctor services in-hours and GPOOH; unscheduled care) in 2015 and 2016.

*Participants*: Aggregated statistics on contacts to NHS111, GPIH and GPOOH were computed based on 13 million respiratory contacts.

*Primary and secondary outcome measures*: Respiratory contact rate to NHS111, GPIH and GPOOH, and non-respiratory contact rate to NHS111 and GPOOH.

*Results:* For all systems more respiratory contacts were for females (RR (95% CI): NHS111:1.59 (1.56-1.62), GPOOH:1.73 (1. 70-1.74), GPIH:1.95 (1.87-2.03)), the those <1 year (NHS11: 37.32 (36.10-38.85), GPOOH: 18.66 (17.78-19.58)), GPIH: 6.21 (5.95-6.48)) and those from more deprived areas (NHS111: 1.75 (1.561-1.95), GPOOH: 2.70 (1.79-4.08)). Elevated contacts were observed for males <5 years for respiratory contacts compared to females <5 years. All services showed similar healthcare-seeking behaviours between respiratory and non-respiratory contacts.



For peer review only - http://bmjopen.bmj.com/site/about/guidelines.xhtml

*Conclusion:* When contact rates in services that captured a more of the disease burden are explored, the demographic patterns are similar to those observed in acute systems. Comparable results were observed between respiratory and non-respiratory contacts suggesting that when a wider spectrum of disease is explored, demographic and socioeconomic factors may be the strongest influencers of healthcare-seeking behaviours. Higher contact rates in males <5 years for both respiratory and non-respiratory contacts requires further research.

**Article Summary**

**Strengths and limitations of this study**

- One of the largest multiservice, observational studies ever undertaken of over 13 million respiratory-related contacts to explore the demographic and socioeconomic patterns of healthcare-seeking behaviour for respiratory symptoms in England.
- Data from three commonly used community healthcare services: NHS111; a telehealth helpline, and GPIH; family doctor services in-hours and GPOOH; unscheduled care, were included in the study, allowing for a comprehensive analysis of respiratory health care seeking behaviour in the community.
- For NHS111 and GPOOH services, non-respiratory contacts were explored to allow us to identify similarities and differences between demographic and socioeconomic patterns in health-care seeking behaviours for respiratory and non-respiratory illnesses.
- Data was obtained from three real-time community healthcare services and due to this did not contain information of co-morbidities. This could not be accounted for in the analysis.



For peer review only - http://bmjopen.bmj.com/site/about/guidelines.xhtml

## Introduction:

Acutely presenting respiratory disease, henceforth referred to as respiratory disease, including upper and lower respiratory tract infections and asthma, have a substantial impact on individual health and healthcare systems. Globally, lower respiratory tract infections (LRTI) were the sixth leading cause of death, causing over 2.3 million deaths in 2016 (1). Upper respiratory tract infections (URTI) have a substantial impact on health burden with over 4.7 million disability-adjusted-life years (DALYs) globally in 2016 (2). Although asthma is a chronic condition, it often presents with acute exacerbations. Worldwide, as of 2015, around 358 million people were living with asthma (3).

In the Global Burden of Disease Study 2010 (4), the UK had the second highest number of age-standardised DALYs due to LRTI and asthma out of 19 other high-income countries (5). In comparison to 15 other European countries, the UK had one of the highest mortality rates due to respiratory infections (6). In the UK, 15.8% of the population are predicted to develop asthma in their lifetime (6). LRTI and URTI are estimated to cost over £1.7 billion, and asthma over £3 billion annually (7).

To help develop effective interventions it is necessary to understand who is most at risk. Deprivation has been linked to morbidity and mortality of asthma and respiratory infections (8–11). Factors attributed to higher rates of respiratory diseases in more deprived areas include higher smoking rates (12), higher levels of pollution (13), and poor quality housing (14). Females have higher rates of presentation with URTI compared to males (15). However, males succumb more to LRTI, which are more severe than URTI and lead to higher mortality rates (15). Gender differences in respiratory diseases are affected by age, with male children more prone to illness, hospitalisation, and death due to respiratory diseases (16). This age-gender interaction has been observed in asthma, with prepubescent boys more likely to develop asthma; by early puberty the prevalence equalises (17–19).

Here, we focus on the social patterning of respiratory disease. Unlike previous studies, which often focus on acute measures of respiratory disease, such as hospitalisations, we utilise data sources that

For peer review only



For peer review only - http://bmjopen.bmj.com/site/about/guidelines.xhtml

should capture more disease burden. Our data is defined as non-specific, pre-diagnostic-syndromic data which we use as a proxy for disease (20).

Syndromic data has previously been used to investigate associations between demographics, deprivation and disease. Todkill et al. (21), used syndromic data from family doctors presentations for allergic rhinitis to investigate socioeconomic associations. Higher rates of allergic rhinitis were observed in females, children and those from more deprived areas. Lusignan et al. (22), used family-doctor coded diagnoses to conduct a large-scale investigation on the impact of age, gender and deprivation on respiratory illnesses in England. Those in the most deprived quintile had a higher probability of presenting with common cold. Nilsson et al. (23), investigated associations between antibiotic prescriptions for penicillin-non-susceptible Streptococcus pneumonia (PNSP) and deprivation in Malmö, Sweden. Although deprivation was not linked with higher rates of PNSP, higher deprivation was associated with increased rates of antibiotic prescribing.

One issue with the use of syndromic data in such studies is the difficulty in ascertaining how much of any associations arise from patterns of healthcare-seeking behaviour or disease incidence. This situation could be explored by comparison of results to all healthcare-seeking behaviours, but this appears absent in the literature. Furthermore, most studies utilise a single source of syndromic data making it difficult to know whether the results are specific to a service.

**Aims:**

This study aims to:

1. Explore associations between respiratory-related contacts to three healthcare services; telehealth, family doctors and unscheduled care, and age, gender and ecological measures of deprivation in England.
2. Compare these results to all non-respiratory contacts to identify whether associations are specific to respiratory disease.



For peer review only - http://bmjopen.bmj.com/site/about/guidelines.xhtml

## Methods:

### Data Collection

#### *Syndromic surveillance data*

Public Health England (PHE) coordinates a national programme of syndromic surveillance using multiple systems: a telehealth service (NHS111) which operates continuously all year; a family doctor service (GPIH) that operates during weekday working hours, and an out-of-hours family physician system (GPOOH). These services run as part of the National Health Service (NHS) which is universal and free at the point of delivery.

Syndromic data comprised of annual counts, between 1 January 2015 and 31 December 2016, of respiratory and non-respiratory contacts with the three syndromic surveillance systems coordinated by PHE.  Syndromic indicators classified as respiratory are presented in Table 1, and previous studies have demonstrated the association between respiratory disease and these syndromic indicators (24,25).  Non-respiratory counts were not available for GPIH.

Data were obtained at the finest geographical level available; postcode district (PD) (e.g. SW1) for NHS111 and GPOOH, and PHE Centre (e.g. London) for GPIH. In England, there are 2,234 PDs with, on average, 25,660 (142-162,266) residents. There are nine PHE Centres with an average of 6,181,375 (2,644,727-9,080,825) residents. Count data were subdivided by age group: under-1, 1-4, 5-14, 15-44, 45-64, 65-74 and over-75 years; by gender (male/female), year (2015/2016); and by geographic location.

#### *Demographic data*

PD population, obtained from the 2011 census (26), grouped by age and gender, was used as a denominator in the analysis of NHS111 and GPOOH contacts. GPIH populations were derived from the sum of registered populations at each participating GPIH practice.



For peer review only - http://bmjopen.bmj.com/site/about/guidelines.xhtml

***Independent variables***

The Index of Multiple Deprivation (IMD) (27) was used as an area-level measure of deprivation. IMD scores were obtained at Lower Layer Super Output Area (LSOA); a weighted mean for each PD was calculated using the proportion of LSOA population in each PD. Weighted means were divided into quintiles from most (1) to least (5) deprived.

**Data Cleaning and Exploration**

NHS111 respiratory contacts from Essex in 2015 & 2016 and Norfolk in 2016 were excluded because syndromic data was unavailable. Syndromic surveillance coverage maps of GPOOH were obtained at the upper tier local authority (UTLA) geographical level, and data were excluded from any UTLA where the PHE programme received little or no syndromic data.

For all systems, data were excluded if location, age or gender of healthcare seeker was unknown (Table 2). PD that were demarcated for large organisations (e.g. Heathrow Airport), or had less than 200 residents were excluded from analysis as their small populations or unique nature are unlikely to produce reliable estimates. PD that overlap borders with Scotland and Wales were excluded. For each system, contact rates were mapped for both study years to visualise spatial variation (Appendix 1).

**Statistical Analysis**

To measure the relationship between the dependent and independent variables generalised linear mixed models (GLMMs) were used for NHS111 and GPOOH, while a generalised linear model (GLM) was used for GPIH. GPIH data was modelled using both GLM and GLMM methods, however the GLM provided a better model fit (Appendix 3). The study design accounted for the hierarchical structure of the data for each system by including PD and UTLA as random effects in the NHS111 and GPOOH models. Upper-tier local authority (UTLA) was also included as a random effect to account for similar characteristics of neighbouring PD, to reduce the effect of spatial autocorrelation, and UTLAs are responsible for services that might influence presentations (map of UTLA overlaid PD in Appendix 2). Where a PD was located in more than one UTLA, the largest PD area was



For peer review only - http://bmjopen.bmj.com/site/about/guidelines.xhtml

allocated. PHE Centre was included as a fixed effect in the GPIH model. To account for population differences at the geographical level, the logarithm of the population plus one was included as a model offset. We explored Poisson and negative binomial model specifications to account for potential over-dispersion in the syndromic data. Over-dispersion was tested by comparing the sum of squared Pearson residuals to the residual degrees of freedom. Models with over-dispersion statistics <1.5 were deemed acceptable (28). Wald tests were used to determine the overall significance of variables. A categorical variable for year was included in the model to account for inter-annual variation. Percentage urban area was included to account for differences in healthcare-seeking behaviour or disease risk related to urbanicity. Two-way interactions between age and gender, and age and deprivation were investigated in the NHS111 and GPOOH models. Highly aggregated deprivation data at PHE centre meant only age and gender interactions were investigated in the GPIH model. Rate ratios with 95% confidence intervals were estimated. Model overfitting was assessed using k-fold cross-validation (Appendix 4). Model predictions were standardised to zero mean and unit variance by subtracting the mean of the predictions from each predicted value and then dividing by their standard deviation to allow a comparison of trends between the models.

All analyses were conducted in R (29) and models were specified using the glmmTMB (30) and MASS (31) packages.



For peer review only - http://bmjopen.bmj.com/site/about/guidelines.xhtml

## Results:

### Data and model selection

Table 1 indicates that relatively few contacts were excluded and any data issues observed in the datasets were because of the passive reporting to the surveillance systems and not due to disruption of healthcare service. Table 2 demonstrates that in total 21,242,154 contacts to NHS111 were included in the analysis, of which 8.10% (n = 1,721,034) were respiratory contacts. 6,937,657 GPOOH contacts were included in the analysis of which 22.53% (n = 1,562,883) were respiratory contacts. Total number of contacts were not available for GPIH, but 10,310,626 respiratory contacts were included in the study for GPIH data.

Two model distributions were considered: Poisson, and negative binomial, with models selected by considering over-dispersion of the data. Negative binomial models handled the over-dispersed data best in all five models compared to the Poisson distribution (descriptions of model fit in Appendix 5).

### Multivariable Analysis

The results of the multivariable analysis for each model are visualised in Figures 1 to 3 alongside the overall significance of each model. The full table of results is presented in Appendix 5.

The main effects indicate that age (Figure 1a) and gender (Figure 1b) have significant influences on contacts to each service and visually the differences between independent variables appears similar between respiratory and non-respiratory contacts and across all systems, with contacts significantly higher the youngest age groups. There are significantly more contacts regarding females in both the respiratory (RR: 1.59; 95%CI: 1.56–1.62) and non-respiratory (RR 1.75; 95%CI: 1.72–1.77) NHS111 models, with similar results in the GPOOH and GPIH models.  Deprivation (Figure 1c) as a main effect is significant in both NHS111 models, which found there are 75% more of contacts in the most deprived areas compared to the least deprived (IMD quintile 1 vs 5). Similar rate ratios for the least deprived areas were observed in both the respiratory (RR: 1.75; 95%CI: 1.56– 1.95) and non-respiratory NHS111 models (RR 1.81; 95%CI: 1.66–1.96). Deprivation was significant in both the respiratory and non-respiratory GPOOH models, with more contacts in the most-deprived areas



For peer review only - http://bmjopen.bmj.com/site/about/guidelines.xhtml

compared to the least deprived (respiratory RR: 2.70; 95%CI: 1.79-4.08, non-respiratory RR: 2.70; 95%CI: 1.89-3.85)

Age-gender interactions (Figure 2) show similar trends across system types and when compared to non-respiratory contacts. Overall, there are more female contacts; but in all models, in the younger age groups (under-1s and 1-4-year-olds) more male contacts were observed.

Age-deprivation interactions (Figure 3) were investigated for NHS111 and GPOOH. In both respiratory and non-respiratory contacts to NHS111 (Figure 3a), the trends suggest there are more contacts in the most deprived quintiles across all ages. For respiratory contacts, there is a stronger trend with deprivation in the under-1, 1-4 and over-75-year age groups, and a weaker trend in the remaining age groups. This NHS111 trend is strong across all age groups in the non-respiratory model, particularly in the age group over-75 years. When broken down by age, there was a linear trend with deprivation and contacts to GPOOH (Figure 3b), with more contacts in most-deprived areas.

For peer review only



For peer review only - http://bmjopen.bmj.com/site/about/guidelines.xhtml

## Discussion:

### Impact

We present a large-scale analysis of over 13 million respiratory-related contacts to three healthcare services in England. These services deliver healthcare to patients at the community level, and may be better indicators of overall disease patterns as opposed to the relatively small proportion that appear in hospitalisation or laboratory-based surveillance. Access to these large syndromic datasets has allowed a comprehensive analysis of the demographics using of each service and the impact deprivation has on healthcare contacts. It has also allowed the interactions between these factors to be explored. By analysing respiratory and non-respiratory contacts, we were able to identify differences and similarities in usage patterns. Analysing NHS111 and GPOOH contacts at PD, a small level of geography, we explored deprivation patterns to give a thorough analysis of social patterning of factors associated with respiratory contacts.

### Main Findings and Comparison to Literature

In all three systems (NHS111, GPOOH and GPIH), there were more respiratory and non-respiratory contacts in females. Previous research indicates that women are more likely to seek healthcare than men, even when female-specific concerns are accounted for (32,33). In terms of age, contacts were highest in under-1, followed by the 1-4 and the over-75 age groups. These trends were observed across all three systems, and fit with previous research (34,35).

When interactions between age and gender were explored, we found that more presentations were of males in the two youngest age groups for both respiratory and non-respiratory illnesses; this effect was observed across all systems. Previous studies have reported that male children have higher rates of respiratory illnesses (17,34,36). Lusignan et al. (36), observed a higher incidence of family doctors contacts in males under 15 years due to LRTI compared to females of the same age, with the largest gender difference in the youngest age group (under-1s). This study also noted a higher contact rate in males under 15 years due to asthma. The reasons for which are unclear, but immunologic, genetic and biological differences are thought to increase risk, suggesting that the excess in male children is due to genuine predisposition to respiratory illness rather than sociological factors (16). This excess in male



For peer review only - http://bmjopen.bmj.com/site/about/guidelines.xhtml

children was also observed in our data for non-respiratory contacts. The reasons for this excess of non-respiratory contacts in male children is unclear. These findings suggest that there are similar drivers to presentations in male children presenting for all illness types rather than with just respiratory illnesses, whether this be due to differences in healthcare-seeking behaviours or disease aetiology. Earp et al (2019) observed that adults perceived that male children (aged 5-years) experienced more pain compared to females of the same age, despite the same clinical circumstances and identical pain behaviour (37). The Intestinal Infectious Disease (IID) Study observed a higher rate of IID in the community in females under 1-year compared to males, but a higher rate of presentations to family doctors of males compared to females of the same age (38). This observation suggests that either males were more likely to have cases of IID, which required medical intervention, or health-care was more likely to be sought for males compared to females of the same age. There could be gender differences in the perception of ill health in young children from parental caregivers, which could influence health-care seeking behaviour, although this requires more research.

Deprivation as a main effect was significant in both the NHS111 and GPOOH models, with a greater risk of respiratory and non-respiratory contacts in those from more deprived areas. Similar results were found when looking at gastrointestinal illness contacts to NHS111 and deprivation, with more contacts in the most deprived areas (39). Kelly et al. (40), observed higher attendances from those from the most deprived areas, to GPOOH services. One of the relatively unique elements of our work was the exploration of the interactions between deprivation and age. For NHS111, deprivation appears to have a greater impact on non-respiratory contacts than on respiratory contacts. The exception is in the youngest age bracket, where deprivation has a similarly high impact on both respiratory and non-respiratory contacts, with more contacts in most-deprived areas. A similar pattern emerged for GPOOH where there was more contact in the most derived areas; however, this trend was similar across age groups and in respiratory and non-respiratory contacts.

**Limitations**

Although the surveillance of these three services allows us to observe a large number of healthcare contacts, these are working surveillance datasets from real-time surveillance system, with periods



For peer review only - http://bmjopen.bmj.com/site/about/guidelines.xhtml

where data is not received. The large numbers included in the study meant that socioeconomic and demographic patterns could still be observed and omissions are only likely to bias estimates if correlated with independent variables. Coverage issues will increase the uncertainty of our estimates, which is particularly evident in the wide confidence intervals observed in the GPOOH results. The analysis presented here is exploring indicators of disease as opposed to actual disease. Therefore, the analyses are reliant on accurate classifications of disease indicators. NHS111, GPIH and GPOOH services have different symptom coding systems, the definition of respiratory and non-respiratory may differ between systems. The deprivation measures used were composites of multiple factors that contribute to deprivation and were ecological in nature, are subject to the ecological fallacy whereby the associations found at the area level may not hold at the individual level. Data obtained from the surveillance systems did not contain information on co-morbidities or ethnicity, therefore these factors could not be accounted for in the analysis.

**Conclusion**

This large-scale study highlights the effects of age, gender and deprivation on healthcare-seeking behaviours for respiratory diseases, with more healthcare contacts from females, the young and old and those from more deprived areas. Similar patterns were observed across the three services, which were in agreement with the literature. Comparable results were also observed between respiratory and non-respiratory contacts suggesting that even when a wider spectrum of disease is explored, demographic and socioeconomic factors may be the strongest influencers of healthcare-seeking behaviours. When broken down by age there were more contacts regarding male children under-5 years compared to females. This trend was observed in both respiratory and non-respiratory contacts indicating there are more healthcare-seeking behaviours in male children across a range of disease types. These findings could be influenced by sociological factors as well as disease aetiology and requires further research. Further research is required to understand the role that age, gender and deprivation has on healthcare-seeking behaviours.



For peer review only - http://bmjopen.bmj.com/site/about/guidelines.xhtml

Table 1. Number of contacts excluded from study by reason for exclusion.

| | NHS111 (% of total) | GPOOH (% of total) |
|---|---|---|
| Total number provided | 21,905,099 | 9,623,939 |
| **Reason for exclusion** | | |
| No valid postcode provided/Not in England | 613,495 (2.9) | 92,815 (9.6) |
| No gender given | 9,536 (0.04) | 12,312 (0.13) |
| No age given | - | 1,601 (0.017) |
| Postcode District with < 200 population | 44 (0.0002) | 12 (0.0001) |
| Overlapping borders with Scotland or Wales | 17,072 (0.08) | 3,795 (0.04) |
| Large area users/City centres | 1,517 (0.007) | 2,379 (0.03) |
| Poor coverage/Data Issues | 21,281 (0.1) | 1,010,485 (10.50) |
| **Total excluded** | **662,945 (3.02)** | **1,123,399 (11.67)** |

[+]GPIH had no exclusions

Table 2. Indicators for respiratory diseases for each syndromic surveillance system, 1st January 2015 to 31st December 2016.

| System monitored by syndromic surveillance | Routine syndromic indicators included in Respiratory Indicator for this study | Total number of contacts (T) | Total number of respiratory contacts (R) | T-R (non-respiratory) |
|---|---|---|---|---|
| NHS111 | "Cold/flu", "cough" and "difficulty breathing" | 21,242,154 | 1,721,034 | 19,521,120 |
| GPIH [+] | "Upper and lower respiratory tract infections" and "asthma" | –- | 10,310,626 | – |
| GPOOH | "Acute respiratory infection" "asthma", "wheeze" and "difficulty breathing" | 8,500,540 | 1,562,883 | 6,937,657 |

[+] Total number of contacts to GPIH services not available.



For peer review only - http://bmjopen.bmj.com/site/about/guidelines.xhtml

For peer review only

**Acknowledgements**

The authors acknowledge technical support from Paul Loveridge, Ana Soriano, and Helen Hughes (PHE Real-time Syndromic Surveillance Team) and support from: NHS 111 and NHS Digital for their assistance and support with the NHS 111 system; OOH providers submitting data to the GPOOH and Advanced Heath & Care; TPP and participating SystmOne practices and University of Nottingham, ClinRisk, EMIS and EMIS practices submitting data to the QSurveillance database.

KEM, FCG, PRH, AJE, GES and IRL are supported by the National Institute for Health Research Health Protection Research Unit (NIHR HPRU) in Emergency Preparedness and Response at King's College London in partnership with PHE. The views expressed are those of the author(s) and not necessarily those of the NHS, the NIHR, the Department of Health or Public Health England.

**Data Statement**

Sharing: No additional data available.

**Ethics**

Ethical approval for this work was not required. This study was undertaken as part of the national surveillance functions of Public Health England (PHE). The anonymised, aggregated health data used in this study, were routinely collected as part of the public health function of PHE.

**Funding**

KEM is supported by the National Institute for Health Research and University of East Anglia

FCG, PRH, AJE, GES and IRL are supported by the National Institute for Health Research

**Patient and Public Involvement**

None

**Conflicts of Interest**

None Declared

For peer review only

## References


1. Troeger C, Blacker B, Khalil IA, Rao PC, Cao J, Zimsen SRM, et al. Estimates of the global, regional, and national morbidity, mortality, and aetiologies of lower respiratory infections in 195 countries, 1990–2016: a systematic analysis for the Global Burden of Disease Study 2016. The Lancet Infectious Diseases. 2018 Nov 1;18(11):1191–210.

2. Hay SI, Abajobir AA, Abate KH, Abbafati C, Abbas KM, Abd-Allah F, et al. Global, regional, and national disability-adjusted life-yea rs (DALYs) for 333 diseases and injuries and healthy life expectancy (HALE) for 195 countries and territories, 1990–2016: a systematic analysis for the Global Burden of Disease Study 2016. The Lancet. 2017 Sep 16;390(10100):1260–344.

3. Soriano JB, Abajobir AA, Abate KH, Abera SF, Agrawal A, Ahmed MB, et al. Global, regional, and national deaths, prevalence, disability-adjusted life years, and years lived with disability for chronic obstructive pulmonary disease and asthma, 1990–2015: a systematic analysis for the Global Burden of Disease Study 2015. The Lancet Respiratory Medicine. 2017 Sep 1;5(9):691–706.

4. Global Burden of Disease Collaborative Network. Global Burden of Disease Study 2010 (GBD 2010) Results by Risk Factor 1990-2010. Institute for Health Metrics and Evaluation (IHME), Seattle, USA; 2012.

5. Murray CJ, Richards MA, Newton JN, Fenton KA, Anderson HR, Atkinson C, et al. UK health performance: findings of the Global Burden of Disease Study 2010. The Lancet. 2013 Mar 23;381(9871):997–1020.

6. Mukherjee M, Stoddart A, Gupta RP, Nwaru BI, Farr A, Heaven M, et al. The epidemiology, healthcare and societal burden and costs of asthma in the UK and its member nations: analyses of standalone and linked national databases. BMC Medicine. 2016 Aug 29;14(1):113.

7. Trueman D, Woodcock F, Hancock E. Estimating the economic burden of respiratory illness in the UK [Internet]. British Lung Foundation; 2017. Available from: https://www.probonoeconomics.com/sites/default/files/files/British%20Lung%20Foundation%20full%20report%2015032017_0.pdf

8. Sallakh MAA, Rodgers SE, Lyons RA, Sheikh A, Davies GA. Socioeconomic deprivation and inequalities in asthma care in Wales. The Lancet. 2017 Nov 1;390:S19.

9. Jordan R, Verlander N, Olowokure B, Hawker JI. Age, sex, material deprivation and respiratory mortality. Respir Med. 2006 Jul;100(7):1282–5.

10. Riaz SP, Horton M, Kang J, Mak V, Lüchtenborg M, Møller H. Lung cancer incidence and survival in England: an analysis by socioeconomic deprivation and urbanization. J Thorac Oncol. 2011 Dec;6(12):2005–10.

11. Roberts SE, Button LA, Hopkin JM, Goldacre MJ, Lyons RA, Rodgers SE, et al. Influence of social deprivation and air pollutants on serious asthma. European Respiratory Journal. 2012 Sep 1;40(3):785–8.

12. Newton JN, Briggs ADM, Murray CJL, Dicker D, Foreman KJ, Wang H, et al. Changes in health in England, with analysis by English regions and areas of deprivation, 1990–2013: a systematic analysis for the Global Burden of Disease Study 2013. The Lancet. 2015 Dec 5;386(10010):2257–74.


For peer review only

13. Sofianopoulou E, Rushton SP, Diggle PJ, Pless-Mulloli T. Association between respiratory prescribing, air pollution and deprivation, in primary health care. J Public Health (Oxf). 2013 Dec;35(4):502–9.

14. Gibson M, Petticrew M, Bambra C, Sowden AJ, Wright KE, Whitehead M. Housing and health inequalities: a synthesis of systematic reviews of interventions aimed at different pathways linking housing and health. Health Place. 2011 Jan;17(1):175–84.

15. Falagas ME, Mourtzoukou EG, Vardakas KZ. Sex differences in the incidence and severity of respiratory tract infections. Respir Med. 2007 Sep;101(9):1845–63.

16. Liptzin DR, Landau LI, Taussig LM. Sex and the lung: Observations, hypotheses, and future directions. Pediatr Pulmonol. 2015 Dec;50(12):1159–69.

17. Vink NM, Postma DS, Schouten JP, Rosmalen JGM, Boezen HM. Gender differences in asthma development and remission during transition through puberty: the TRacking Adolescents' Individual Lives Survey (TRAILS) study. J Allergy Clin Immunol. 2010 Sep;126(3):498-504.e1-6.

18. Almqvist C, Worm M, Leynaert B, working group of GA2LEN WP 2.5 Gender. Impact of gender on asthma in childhood and adolescence: a GA2LEN review. Allergy. 2008 Jan;63(1):47–57.

19. Bundle N, Verlander NQ, Morbey R, Edeghere O, Balasegaram S, Lusignan S de, et al. Monitoring epidemiological trends in back to school asthma among preschool and school-aged children using real-time syndromic surveillance in England, 2012–2016. J Epidemiol Community Health. 2019 Sep 1;73(9):825–31.

20. Triple S Project. Assessment of syndromic surveillance in Europe. The Lancet. 2011 Dec 2;378(9806):1833–4.

21. Todkill D, Hughes HE, Elliot AJ, Morbey RA, Edeghere O, Harcourt S, et al. An Observational Study Using English Syndromic Surveillance Data Collected During the 2012 London Olympics – What did Syndromic Surveillance Show and What Can We Learn for Future Mass-gathering Events? Prehospital and Disaster Medicine. 2016 Jan;1–7.

22. Lusignan S de, Correa A, Pathirannehelage S, Byford R, Yonova I, Elliot AJ, et al. RCGP Research and Surveillance Centre Annual Report 2014–2015: disparities in presentations to primary care. Br J Gen Pract. 2017 Jan 1;67(654):e29–40.

23. Nilsson P, Laurell MH. Impact of socioeconomic factors and antibiotic prescribing on penicillin- non-susceptible Streptococcus pneumoniae in the city of Malmö. Scandinavian Journal of Infectious Diseases. 2005 Jan 1;37(6–7):436–41.

24. Morbey RA, Harcourt S, Pebody R, Zambon M, Hutchison J, Rutter J, et al. The burden of seasonal respiratory infections on a national telehealth service in England. Epidemiol Infect. 2017 Jul;145(9):1922–32.

25. Morbey RA, Elliot AJ, Harcourt S, Smith S, de Lusignan S, Pebody R, et al. Estimating the burden on general practitioner services in England from increases in respiratory disease associated with seasonal respiratory pathogen activity. Epidemiol Infect. 2018 Aug;146(11):1389–96.

For peer review only

26. Office for National Statistics, National Records of Scotland, Northern Ireland Statistics and Research Agency. 2011 Census aggregate data. 2017;UK Data Service (Edition: February 2017).

27. Smith T, Noble M, Noble S, Wright G, McLennan D, Plunkett E. English indices of deprivation 2015: technical report. London: Department for Communities and Local Government; 2015 Sep.

28. Payne EH, Gebregziabher M, Hardin JW, Ramakrishnan V, Egede LE. An empirical approach to determine a threshold for assessing overdispersion in Poisson and negative binomial models for count data. Communications in Statistics: Simulation and Computation. 2018 Jul;47(6):1722–1738.

29. R Core Team. R: A language and environment for statistical computing. R Foundation for Statistical Computing [Internet]. Vienna, Austria; 2017. Available from: https://www.R-project.org/

30. Brooks ME, Kristensen K, van Benthem KJ, Magnusson A, Berg CW, Nielsen A, et al. glmmTMB Balances Speed and Flexibility Among Packages for Zero-inflated Generalized Linear Mixed Modeling. The R Journal. 2017;9(2):378–400.

31. Venables WN, Ripley BD. Modern Applied Statistics with S [Internet]. Fourth. New York: Springer; 2002. Available from: http://www.stats.ox.ac.uk/pub/MASS4

32. Manierre MJ. Gaps in knowledge: tracking and explaining gender differences in health information seeking. Soc Sci Med. 2015 Mar;128:151–8.

33. Thompson AE, Anisimowicz Y, Miedema B, Hogg W, Wodchis WP, Aubrey-Bassler K. The influence of gender and other patient characteristics on health care-seeking behaviour: a QUALICOPC study. BMC Family Practice. 2016 Mar 31;17(1):38.

34. Liptzin DR, Landau LI, Taussig LM. Sex and the lung: Observations, hypotheses, and future directions. Pediatric Pulmonology. 2015 Dec 1;50(12):1159–69.

35. Salisbury C, Trivella M, Bruster S. Demand for and supply of out of hours care from general practitioners in England and Scotland: observational study based on routinely collected data. BMJ. 2000 Mar;320(7235):618–621.

36. de Lusignan S, Correa A, Pebody R, Yonova I, Smith G, Byford R, et al. Incidence of Lower Respiratory Tract Infections and Atopic Conditions in Boys and Young Male Adults: Royal College of General Practitioners Research and Surveillance Centre Annual Report 2015-2016. JMIR Public Health Surveill. 2018 Apr 30;4(2):e49.

37. Earp BD, Monrad JT, LaFrance M, Bargh JA, Cohen LL, Richeson JA. Featured Article: Gender Bias in Pediatric Pain Assessment. Journal of Pediatric Psychology. 2019 Jan 4;44(4):403–14.

38. Food Standards Agency. A Report of the Study of Infectious Intestinal Disease in England. London HMSO; 2000.

39. Adams NL, Rose TC, Elliot AJ, Smith G, Morbey R, Loveridge P, et al. Social patterning of telephone health-advice for diarrhoea and vomiting: analysis of 24 million telehealth calls in England. Journal of Infection [Internet]. 2018 Sep 26; Available from: http://www.sciencedirect.com/science/article/pii/S0163445318302810

For peer review only

40. Kelly JS, Piercy H, Ibbotson R, Davis SVF. Who attends out-of-hours general practice appointments? Analysis of a patient cohort accessing new out-of-hours units. BMJ Open. 2018 Jun;8(6):e020308.

**Figure Legends**

Figure 1. Standardised main effects of multivariable analysis of respiratory contacts to NHS111, General Practitioner-out-of-hours (GPOOH) and General Practitioner-in-hours (GPIH): a) age group, b) gender and c) deprivation. Overall model significance presented in legend (ns = not significant (overall effect only), * = ≤0.05, ** = ≤0.01, *** = ≤0.001)

Figure 2. Standardised effect sizes for age gender interactions for respiratory and non-respiratory contacts by each system: a) NHS111, b) General Practitioner-in-hours (GPIH) and c) General Practitioner-out-of-hours (GPOOH). Overall model significance presented in legend, (ns = not significant (overall effect only), * = ≤0.05, ** = ≤0.01, *** = ≤0.001)

Figure 3. Standardised effect sizes for age deprivation interactions for respiratory and non-respiratory contacts by each system: a) NHS111, and b) General Practitioner-out-of-hours (GPOOH). Overall model significance presented in legend, (ns = not significant (overall effect only), * = ≤0.05, ** = ≤0.01, *** = ≤0.001)

For peer review only

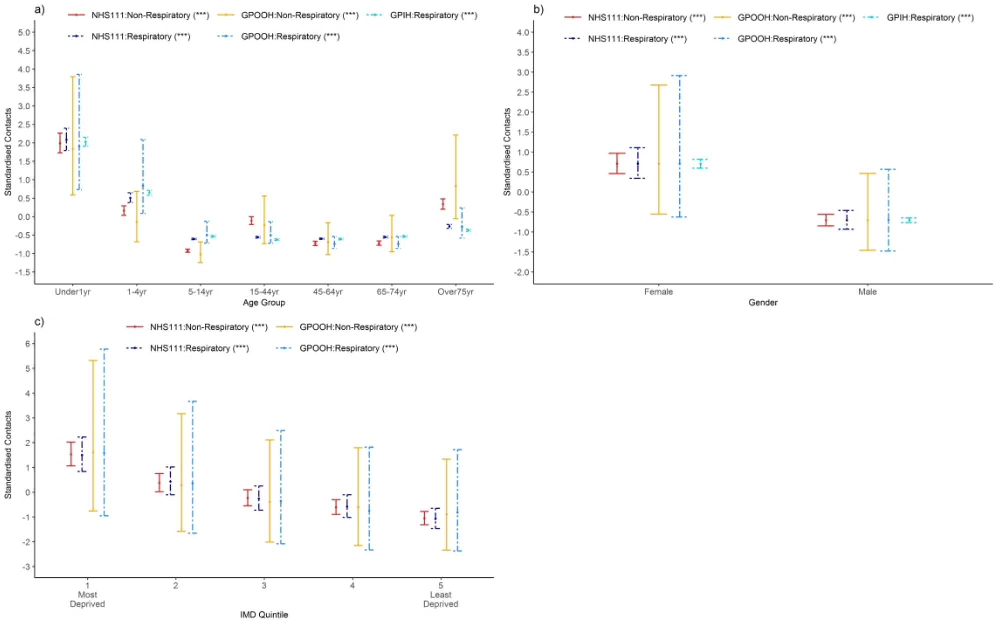


Figure 1. Standardised main effects of multivariable analysis of respiratory contacts to NHS111, General Practitioner-out-of-hours (GPOOH) and General Practitioner-in-hours (GPIH): a) age group, b) gender and c) deprivation. Overall model significance presented in legend (ns = not significant (overall effect only), * = ≤0.05, ** = ≤0.01, *** = ≤0.001)

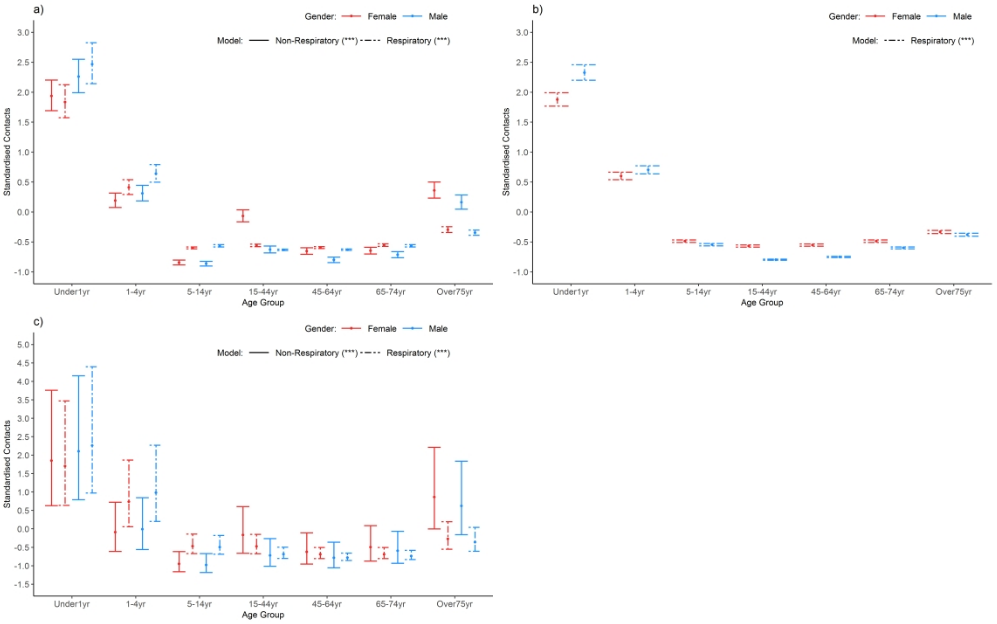


Figure 2. Standardised effect sizes for age gender interactions for respiratory and non-respiratory contacts by each system: a) NHS111, b) General Practitioner-in-hours (GPIH) and c) General Practitioner-out-of-hours (GPOOH). Overall model significance presented in legend, (ns = not significant (overall effect only), * = ≤0.05, ** = ≤0.01, *** = ≤0.001)

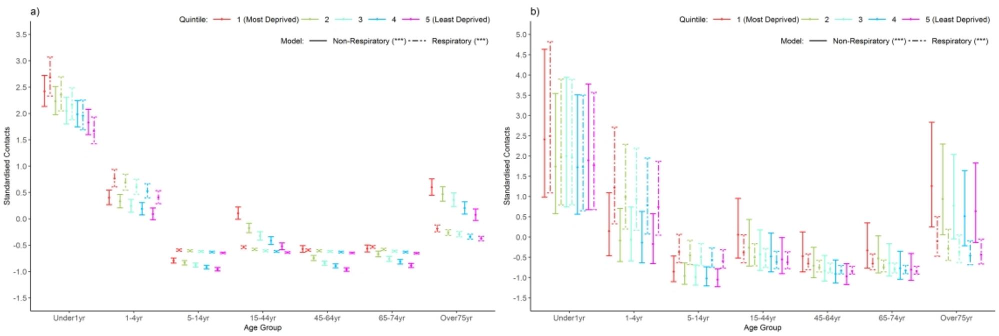


Figure 3. Standardised effect sizes for age deprivation interactions for respiratory and non-respiratory contacts by each system: a) NHS111, and b) General Practitioner-out-of-hours (GPOOH). Overall model significance presented in legend, (ns = not significant (overall effect only), * = ≤0.05, ** = ≤0.01, *** = ≤0.001)

Supplementary Material

Appendix 1.

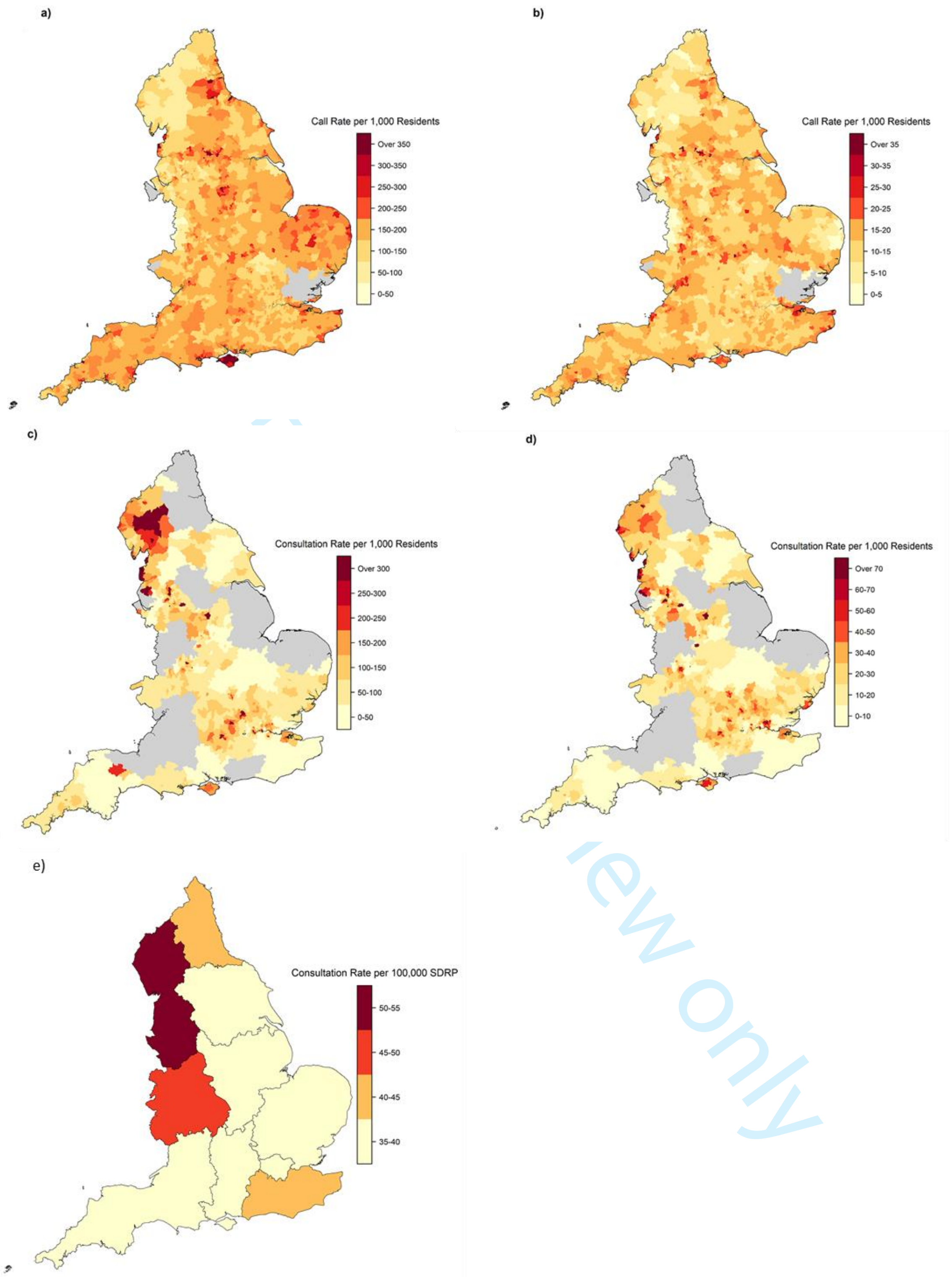


Spatial variation of contacts to three health services in England between 1st January 2015 & 31st December 2016;
a) All non-respiratory calls to NHS111 at PD,
b) Respiratory calls to NHS111 at PD,
c) All non-respiratory consultations to GPOOH services at PD,
d) Respiratory consultations to GPOOH services at PD,
e) Respiratory calls to GPIH services PHE centre. SDPR = Sum of daily registered population
The grey areas indicate the PD that were excluded for the duration of the study period.

Appendix 2

For peer review only - http://bmjopen.bmj.com/site/about/guidelines.xhtml

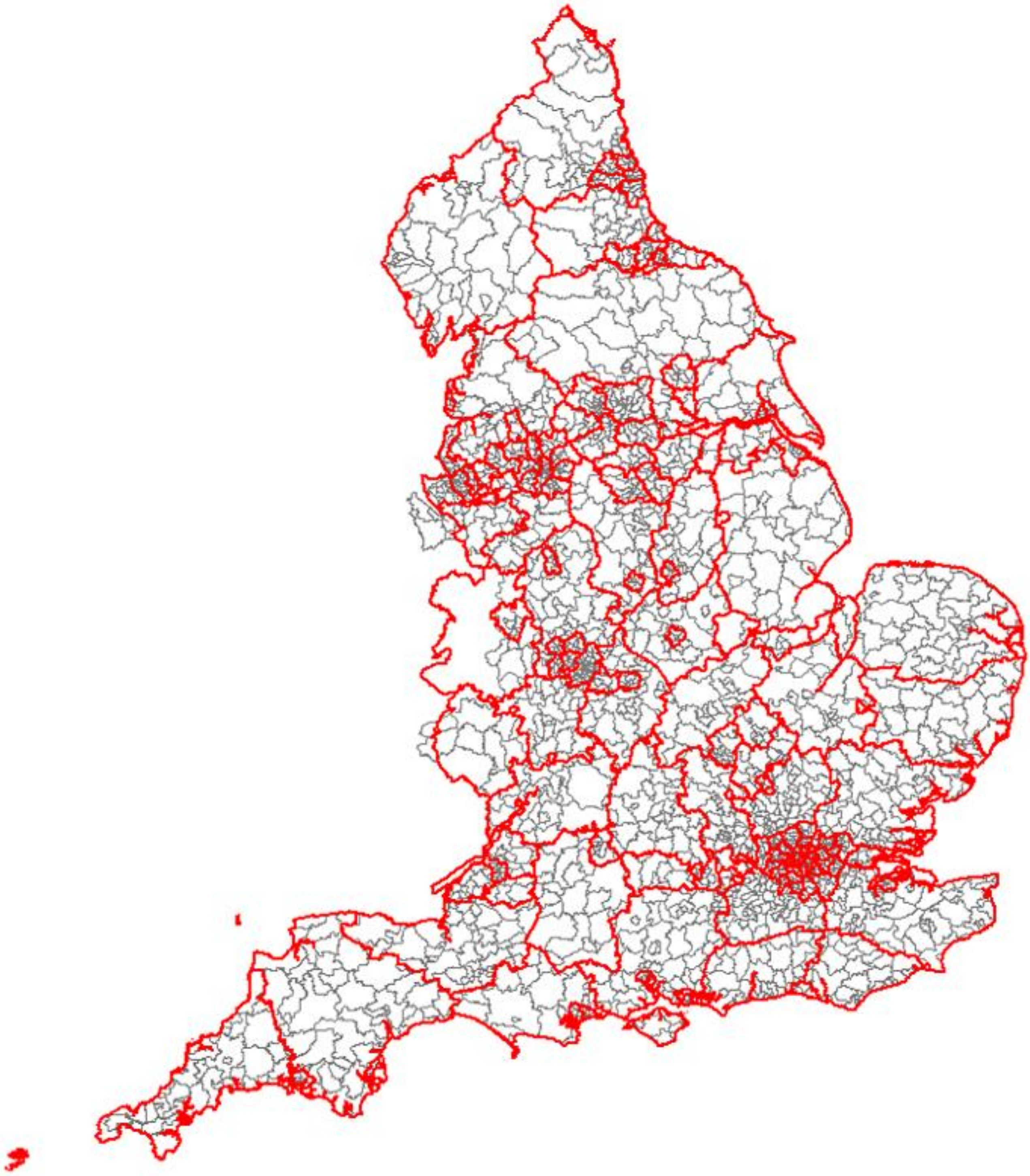

*Upper-tier local authorities (red) overlapped over PD (grey)*

Appendix 3. Comparison between GLM and GLMM results of respiratory calls to GPIH (ns = not significant (overall effect only), * = ≤0.05, ** = ≤0.01, *** = ≤0.001).

| | GLM | | GLMM | |
|---|---|---|---|---|
| Variable | **Rate** Ratio | 95% CI | **Rate** Ratio | 95% CI |
| Intercept | 142.693 | 0.000 –1.455e+16 | 142.264 | 0.000-2.619e+16 |
| Main Effects | | | | |
| Age | | *** | | *** |
| Under 1 year | 6.213 | 5.955 – 6.482*** | 6.213 | 5.950-6.487*** |
| 1-4 years | 3.492 | 3.348 – 3.643*** | 3.492 | 3.450-3.645*** |
| 5-14 years | 1.172 | 1.123 – 1.223*** | 1.172 | 1.123 - 1.223*** |
| 15-44 years | ref | ref | ref | ref |
| 45-64 years | 1.032 | 0.989 – 1.076 | 1.032 | 0.988 - 1.077 |
| 65-74 years | 1.170 | 1.122 – 1.221*** | 1.170 | 1.121 - 1.222*** |
| 75 years and above | 1.500 | 1.438 – 1.564*** | 1.500 | 1.436 - 1.566*** |
| Gender | | | | *** |
| Female | ref | ref | ref | ref |
| Male | 0.513 | 0.492 – 0.535*** | 0.513 | 0.492-0.536*** |
| PHE Centre | - | *** | - | - |
| Year | - | ns | - | ns |
| Interaction Effects | | | | |
| Age:Gender | - | *** | - | *** |
| AIC | 5083.492 | | 5117.537 | |
| Deviance Explained | 0.993 | | 0.992 | |
| Dispersion Statistic | 1.100 | | 1.0332 | |

Appendix 4. Cross validation of final model to assess model overfitting.

| | NSH111 | | GPOOH | | GPIH |
|---|---|---|---|---|---|
| | Respiratory | Non-Respiratory | Respiratory | Non-Respiratory | Respiratory |
| Max number of contacts per location | 391 | 17842 | 2108 | 10175 | 200304 |
| Min number of contacts per location | 0 | 0 | 0 | 0 | 4274 |
| Mean number of contacts per location | 30.9 | 350.3 | 39.2 | 173.9 | 36823.7 |
| Mean of the mean absolute error | 7.02 | 54.6 | 9.5 | 40.5 | 2533.7 |

For peer review only

Appendix 5. Final Model multivariable regression analysis of respiratory and non-respiratory contacts to three healthcare services. Full results and overall significance presented for main effects, and overall significance for interaction terms (ns = not significant, * = ≤0.05, ** = ≤0.01, *** = ≤0.001, x = variable not modelled, - = reference level).

| | NHS 111 | | | | GPOOH | | | | GPIH | |
|---|---|---|---|---|---|---|---|---|---|---|
| | Respiratory Calls | | Non-respiratory Calls | | Respiratory Calls | | Non-respiratory Calls | | Respiratory Calls (PHE Centre | |
| **Variable** | **Rate Ratio** | **95% CI** | **Rate Ratio** | **95% CI** | **Rate Ratio** | **95% CI** | **Rate Ratio** | **95% CI** | **Rate Ratio** | **95% CI** |
| Intercept | 0.005 | 0.004-0.005 | 0.106 | 0.099-0.115 | 0.002 | 0.001-0.003*** | 0.015 | 0.009-0.023*** | 142.693 | 0.000 – 1.455e+16 |
| **Main Effects** | | | | | | | | | | |
| %Urban | 1.002 | 1.002-1.003*** | 1.001 | 1.001-1.002*** | 1.004 | 1.003-1.006*** | 1.004 | 1.003-1.006*** | x | x |
| Age | – | *** | – | *** | – | *** | – | *** | – | *** |
| *Under 1 year* | 37.324 | 36.104-38.850*** | 7.031 | 6.869-7.197*** | 18.661 | 17.783-19.581*** | 7.640 | 7.281-8.018*** | 6.213 | 5.955 – 6.482*** |
| *1 – 4 years* | 16.683 | 16.177-17.206*** | 3.220 | 3.149-3.292*** | 9.822 | 9.400-10.263*** | 2.782 | 2.656-2.913*** | 3.492 | 3.348 – 3.643*** |
| *5 – 14 years* | 1.983 | 1.915-2.054*** | 0.871 | 0.851-0.891*** | 1.879 | 1.794-1.966*** | 0.791 | 0.755-0.829*** | 1.172 | 1.123 – 1.223*** |
| *15-44 years* | ref | ref | ref | ref | ref | ref | ref | ref | ref | ref |
| *45-64 years* | 0.854 | 0.826-0.883*** | 0.670 | 0.656-0.685*** | 0.515 | 0.492-0.540*** | 0.795 | 0.761-0.831*** | 1.032 | 0.989 – 1.076 |
| *65-74 years* | 1.372 | 1.324-1.423*** | 0.912 | 0.892-0.932*** | 0.674 | 0.640-0.709*** | 1.247 | 1.192-1.304*** | 1.170 | 1.122 – 1.221*** |
| *75 years and over* | 3.999 | 3.872-4.130*** | 2.673 | 2.616-2.732*** | 2.500 | 2.388-2.617*** | 4.029 | 3.855-4.211*** | 1.500 | 1.438 – 1.564*** |
| Deprivation Quintile | – | *** | – | *** | – | *** | – | *** | – | – |
| *(Most Deprived) 1* | 1.745 | 1.561-1.949*** | 1.814 | 1.658-1.985*** | 2.704 | 1.792-4.081*** | 2.700 | 1.892-3.853*** | x | x |
| *2* | 1.382 | 1.291-1.480*** | 1.387 | 1.313-1.466*** | 1.654 | 1.326-2.062*** | 1.659 | 1.374-2.003*** | x | x |
| *3* | 1.172 | 1.104-1.245*** | 1.179 | 1.124-1.236*** | 1.318 | 1.089-1.596** | 1.231 | 1.045-1.451* | x | x |

| | | | | | | | | | | |
|---|---|---|---|---|---|---|---|---|---|---|
| *4* | 1.075 | 1.019-1.133** | 1.085 | 1.040-1.132*** | 1.153 | 0.986-1.153 | 1.200 | 1.050-1.372** | x | x |
| *(Least Deprived) 5* | ref | ref | ref | ref | ref | ref | ref | ref | x | x |
| Gender | – | *** | | *** | – | *** | – | *** | – | – |
| *Male* | ref | ref | ref | ref | ref | ref | ref | ref | ref | ref |
| *Female* | 1.588 | 1.558-1.618*** | 1.748 | 1.722-1.774*** | 1.733 | 1.697-1.770*** | 1.711 | 1.665-1.758 | 1.949 | 1.868-2.033*** |
| PHE Centre | – | *** | – | *** | – | *** | – | *** | – | *** |
| Year | – | *** | – | *** | – | *** | – | *** | – | ns |
| **Interaction Effects** | | | | | | | | | | |
| Age:Gender | – | *** | – | ** | – | *** | – | *** | – | *** |
| Age:Deprivation | – | *** | – | *** | – | *** | – | *** | x | x |
| Gender:Deprivation | – | *** | – | ** | – | *** | – | ns | x | x |
| Urbanicity (%):Age | – | *** | – | *** | – | *** | – | *** | x | x |
| Urbanicity(%):Deprivation | – | ns | – | * | – | ** | – | ** | x | x |
| **R-Squared** | | | | | | | | | | |
| **Marginal** | | 0.864 | | 0.813 | | 0.303 | | 0.227 | | 0.993 |
| **Conditional** | | 0.941 | | 0.915 | | 0.959 | | 0.945 | | - |
| **Dispersion statistics** | | 1.1572 | | 1.262 | | 1.088 | | 1.025 | | 1.100 |

iew only

**2STROBE 2007 (v4) Statement—Checklist of items that should be included in reports of *cross-sectional studies***

| Section/Topic | Item # | Recommendation | Reported on page # |
|---|---|---|---|
| **Title and abstract** | 1 | (*a*) Indicate the study's design with a commonly used term in the title or the abstract | 3 |
| | | (*b*) Provide in the abstract an informative and balanced summary of what was done and what was found | 3/4 |
| **Introduction** | | | |
| Background/rationale | 2 | Explain the scientific background and rationale for the investigation being reported | 5/4 |
| Objectives | 3 | State specific objectives, including any prespecified hypotheses | 6 |
| **Methods** | | | |
| Study design | 4 | Present key elements of study design early in the paper | 6/7 |
| Setting | 5 | Describe the setting, locations, and relevant dates, including periods of recruitment, exposure, follow-up, and data collection | 7 |
| Participants | 6 | (*a*) Give the eligibility criteria, and the sources and methods of selection of participants | 7 |
| Variables | 7 | Clearly define all outcomes, exposures, predictors, potential confounders, and effect modifiers. Give diagnostic criteria, if applicable | 7/8/9 |
| Data sources/ measurement | 8* | For each variable of interest, give sources of data and details of methods of assessment (measurement). Describe comparability of assessment methods if there is more than one group | 7/8 |
| Bias | 9 | Describe any efforts to address potential sources of bias | |
| Study size | 10 | Explain how the study size was arrived at | NA – surveillance system used which covers large proportion of population |
| Quantitative variables | 11 | Explain how quantitative variables were handled in the analyses. If applicable, describe which groupings were chosen and why | 8/9 |
| Statistical methods | 12 | (*a*) Describe all statistical methods, including those used to control for confounding | 8/9 |
| | | (*b*) Describe any methods used to examine subgroups and interactions | 8/9 |

For peer review only

| | | | |
|---|---|---|---|
| | | (*c*) Explain how missing data were addressed | 8 & Table 1 on page 15 |
| | | (*d*) If applicable, describe analytical methods taking account of sampling strategy | NA |
| | | (*e*) Describe any sensitivity analyses | 8/9 |
| **Results** | | | |
| Participants | 13* | (a) Report numbers of individuals at each stage of study—eg numbers potentially eligible, examined for eligibility, confirmed eligible, included in the study, completing follow-up, and analysed | 10 & Table 2 on page 15 |
| | | (b) Give reasons for non-participation at each stage | Table 1 on page 15 |
| | | (c) Consider use of a flow diagram | NA |
| Descriptive data | 14* | (a) Give characteristics of study participants (eg demographic, clinical, social) and information on exposures and potential confounders | 10/11 |
| | | (b) Indicate number of participants with missing data for each variable of interest | 10 & Table 1 on page 15 |
| Outcome data | 15* | Report numbers of outcome events or summary measures | 10 |
| Main results | 16 | (*a*) Give unadjusted estimates and, if applicable, confounder-adjusted estimates and their precision (eg, 95% confidence interval). Make clear which confounders were adjusted for and why they were included | 10/11 & Appendix 5 |
| | | (*b*) Report category boundaries when continuous variables were categorized | 10/11 |
| | | (*c*) If relevant, consider translating estimates of relative risk into absolute risk for a meaningful time period | NA |
| Other analyses | 17 | Report other analyses done—eg analyses of subgroups and interactions, and sensitivity analyses | 12 |
| **Discussion** | | | |
| Key results | 18 | Summarise key results with reference to study objectives | 12 |
| Limitations | 19 | Discuss limitations of the study, taking into account sources of potential bias or imprecision. Discuss both direction and magnitude of any potential bias | 14 |
| Interpretation | 20 | Give a cautious overall interpretation of results considering objectives, limitations, multiplicity of analyses, results from similar studies, and other relevant evidence | 12/13/14 |
| Generalisability | 21 | Discuss the generalisability (external validity) of the study results | 14 |
| **Other information** | | | |
| Funding | 22 | Give the source of funding and the role of the funders for the present study and, if applicable, for the original study on which the present article is based | 16 |

For peer review only

*Give information separately for cases and controls in case-control studies and, if applicable, for exposed and unexposed groups in cohort and cross-sectional studies.

**Note:** An Explanation and Elaboration article discusses each checklist item and gives methodological background and published examples of transparent reporting. The STROBE checklist is best used in conjunction with this article (freely available on the Web sites of PLoS Medicine at http://www.plosmedicine.org/, Annals of Internal Medicine at http://www.annals.org/, and Epidemiology at http://www.epidem.com/). Information on the STROBE Initiative is available at www.strobe-statement.org.

For peer review only

BMJ Open

# Demographic and socioeconomic patterns in healthcare-seeking behaviour for respiratory symptoms in England; A comparison with non-respiratory symptoms and between three healthcare services

| | |
|---|---|
| Journal: | *BMJ Open* |
| Manuscript ID | bmjopen-2020-038356.R1 |
| Article Type: | Original research |
| Date Submitted by the Author: | 03-Jul-2020 |
| Complete List of Authors: | Morrison, Kirsty; University of East Anglia, School of Environmental Sciences<br>Colón-González, Felipe ; University of East Anglia, School of Environmental Sciences<br>Morbey, Roger; Public Health England, Real-time Syndromic Surveillance Team<br>Hunter, Paul; University of East Anglia, Norwich Medical School<br>Rutter, Judith; NHS Digital<br>Stuttard, Gareth; NHS England and NHS Improvement<br>de Lusignan, Simon; University of Oxford, Nuffield Department of Primary Care Health Sciences; University of Surrey, Department of Clinical and Experimental Medicine<br>Yeates, Alex; Advanced<br>Pebody, Richard; Public Health England, Influenza and Other Respiratory Virus Section, Immunisation and Countermeasures Division, National Infection Service<br>Smith, Gillian; Public Health England, Real-time Syndromic Surveillance Team; University of East Anglia, School of Environmental Sciences<br>Elliot, Alex; Public Health England, Real-time Syndromic Surveillance Team<br>Lake, Iain; University of East Anglia, School of Environmental Sciences |
| <b>Primary Subject Heading</b>: | Epidemiology |
| Secondary Subject Heading: | Public health, General practice / Family practice |
| Keywords: | Epidemiology < INFECTIOUS DISEASES, STATISTICS & RESEARCH METHODS, Respiratory infections < THORACIC MEDICINE |
| | |

SCHOLARONE™
Manuscripts

*I, the Submitting Author has the right to grant and does grant on behalf of all authors of the Work (as defined in the below author licence), an exclusive licence and/or a non-exclusive licence for contributions from authors who are: i) UK Crown employees; ii) where BMJ has agreed a CC-BY licence shall apply, and/or iii) in accordance with the terms applicable for US Federal Government officers or employees acting as part of their official duties; on a worldwide, perpetual, irrevocable, royalty-free basis to BMJ Publishing Group Ltd ("BMJ") its licensees and where the relevant Journal is co-owned by BMJ to the co-owners of the Journal, to publish the Work in this journal and any other BMJ products and to exploit all rights, as set out in our licence.*

*The Submitting Author accepts and understands that any supply made under these terms is made by BMJ to the Submitting Author unless you are acting as an employee on behalf of your employer or a postgraduate student of an affiliated institution which is paying any applicable article publishing charge ("APC") for Open Access articles. Where the Submitting Author wishes to make the Work available on an Open Access basis (and intends to pay the relevant APC), the terms of reuse of such Open Access shall be governed by a Creative Commons licence – details of these licences and which Creative Commons licence will apply to this Work are set out in our licence referred to above.*

*Other than as permitted in any relevant BMJ Author's Self Archiving Policies, I confirm this Work has not been accepted for publication elsewhere, is not being considered for publication elsewhere and does not duplicate material already published. I confirm all authors consent to publication of this Work and authorise the granting of this licence.*

For peer review only

**Title:** Demographic and socioeconomic patterns in healthcare-seeking behaviour for respiratory symptoms in England; A comparison with non-respiratory symptoms and between three healthcare services

**Authors:**

Kirsty E Morrison[1], Felipe J Colón-González[1], Roger A Morbey[3], Paul R Hunter[2], Judith Rutter[4], Gareth Stuttard[5], Simon de Lusignan[6], Alex Yeates[7], Richard Pebody[8], Gillian Smith[1,3], Alex J. Elliot[3], Iain R Lake[1]

**Author Affiliations**

[1]School of Environmental Sciences, University of East Anglia, Norwich, Norfolk, United Kingdom.
[2]Norwich Medical School, University of East Anglia, Norwich, Norfolk, United Kingdom.
[3] Real-time Syndromic Surveillance Team, Field Service, National Infection Service, Public Health England, Birmingham, United Kingdom.
[4] NHS Digital, Leeds, United Kingdom.
[5] NHS England and NHS Improvement, London, United Kingdom.
[6] Nuffield Department of Primary Care Health Sciences, University of Oxford, Oxford, United Kingdom.
[7] Advanced, Ashford, United Kingdom.
[8] Influenza and Other Respiratory Virus Section, Immunisation and Countermeasures Division, National Infection Service, Public Health England, London, United Kingdom.

**Corresponding Author:**

Kirsty E Morrison,

School of Environmental Sciences,

University of East Anglia,

Norwich Research Park,

NR4 7TJ.

07850752299

k.morrison@uea.ac.uk

Word count: 3,997

**Key Words: Epidemiology; Statistics & Research Methods; Deprivation; Respiratory Infections**



For peer review only - http://bmjopen.bmj.com/site/about/guidelines.xhtml

Author Contributions:

Kirsty E Morrison – Responsible for conception and design of study, analysis and interpretation of data and drafting the manuscript.

Felipe J Colón-González – Significant input in the conception and design of study, analysis and interpretation of data and drafting the manuscript and critically reviewed the final version of the work

Roger A Morbey - Significant input in the conception and design of study, interpretation of data, drafting the manuscript and critically reviewed the final version of the work

Paul R Hunter - Critically reviewed the final version of the work

Judith Rutter - Critically reviewed the final version of the work

Gareth Stuttard - Critically reviewed the final version of the work

Simon de Lusignan - Critically reviewed the final version of the work

Alex Yeates - Critically reviewed the final version of the work

Richard Pebody - Critically reviewed the final version of the work

Gillian Smith - Significant input in the conception and design of study, interpretation of data, drafting the manuscript and critically reviewed the final version of the work

Alex J. Elliot - Significant input in the conception and design of study, interpretation of data, drafting the manuscript and critically reviewed the final version of the work

Iain R Lake - Significant input in the conception and design of study, analysis and interpretation of data and drafting the manuscript and critically reviewed the final version of the work

All authors approved the final version of the manuscript.

For peer review only

## Abstract

*Objective:* This study will analyse respiratory contacts to three healthcare services that capture more of the community disease burden than acute data sources, such as hospitalisations. The objective is to explore associations between contacts to these services and the patient's age, gender, and deprivation. Results will be compared between healthcare services, and with non-respiratory contacts to explore how contacts differ by service and illness. It is crucial to investigate the sociodemographic patterns in healthcare-seeking behaviour to enable targeted public health interventions.

*Design*: Ecological Study

*Setting*: Surveillance of respiratory contacts to three healthcare services in England (NHS111; telehealth helpline, GPIH (General Practitioner In-Hours); family doctor services in-hours and GPOOH (General Practitioner Out-of-Hours); unscheduled care).

*Participants*: Aggregated counts on 13 million respiratory contacts to NHS111, GPIH and GPOOH.

*Outcome measures*: Respiratory contacts to NHS111, GPIH and GPOOH, and non-respiratory contacts to NHS111 and GPOOH.

*Results*: More respiratory contacts were observed for females, with 1.59, 1.73, and 1.95 times the rate of contacts to NHS111, GPOOH and GPIH, respectively. When compared to 15-44 year-olds, there were 37.32, 18.66, and 6.21 times the rate of respiratory contacts to NHS111, GPOOH and GPIH in children <1 year. There were 1.75 and 2.70 times the rate of respiratory contacts in the most deprived areas compared to the least deprived to NHS111 and GPOOH. Elevated respiratory contacts were observed for males <5 years compared to females <5 years. Healthcare-seeking behaviours between respiratory and non-respiratory contacts were similar.

*Conclusion*: When contacts to services that capture more of the disease burden are explored, the demographic patterns are similar to those described in the literature for acute systems. Comparable results were observed between respiratory and non-respiratory contacts suggesting that when a wider spectrum of disease is explored, sociodemographic factors may be the strongest influencers of healthcare-seeking behaviour.


For peer review only



For peer review only - http://bmjopen.bmj.com/site/about/guidelines.xhtml

## Article Summary

### Strengths and limitations of this study

- One of the largest multiservice, observational studies ever undertaken of over 13 million respiratory-related contacts to explore the demographic and socioeconomic patterns of healthcare-seeking behaviour for respiratory symptoms in England.
- Data from three commonly used community healthcare services: NHS111; a telehealth helpline, and GPIH; family doctor services in-hours and GPOOH; unscheduled care, were included in the study, allowing for a comprehensive analysis of respiratory healthcare-seeking behaviour in the community.
- For NHS111 and GPOOH services, non-respiratory contacts were explored to allow us to identify similarities and differences between demographic and socioeconomic patterns in healthcare-seeking behaviours for respiratory and non-respiratory illnesses.
- Data was obtained from the real-time surveillance of three community healthcare services and due to this did not contain information of co-morbidities. Therefore, this could not be accounted for in the analysis.



For peer review only - http://bmjopen.bmj.com/site/about/guidelines.xhtml

## Introduction:

Acutely presenting respiratory diseases, henceforth referred to as respiratory disease, including upper and lower respiratory tract infections and asthma, have a substantial impact on individual health and healthcare systems. Globally, lower respiratory tract infections (LRTI) were the sixth leading cause of death, causing over 2.3 million deaths in 2016 (1). Upper respiratory tract infections (URTI) have a substantial impact on health burden with over 4.7 million disability-adjusted-life years (DALYs) globally in 2016 (2). Although asthma is a chronic condition, it often presents with acute exacerbations. Worldwide, as of 2015, around 358 million people were living with asthma (3).

In the Global Burden of Disease Study 2010 (4), the United Kingdom (UK) had the second highest number of age-standardised DALYs due to LRTI and asthma out of 19 other high-income countries (5). In comparison to 15 other European countries, the UK had one of the highest mortality rates due to respiratory infections (6). In the UK, 15.8% of the population are predicted to develop asthma in their lifetime (6). LRTI and URTI are estimated to cost the UK over £1.7 billion, and asthma over £3 billion annually (7).

To help develop effective interventions it is necessary to understand who is most at risk. Deprivation has been linked to morbidity and mortality of asthma and respiratory infections (8–11). Factors attributed to higher rates of respiratory diseases in more deprived areas include higher smoking rates (12), higher levels of pollution (13), and poor-quality housing (14). Females have higher rates of presentation with URTI compared to males (15). However, males succumb more to LRTI, which are more severe than URTI and lead to higher mortality rates (15). Gender differences in respiratory diseases are affected by age, with male children more prone to illness, hospitalisation, and death due to respiratory diseases (16). This age-gender interaction has been observed in asthma, with prepubescent boys more likely to develop asthma; by early puberty the prevalence equalises (17–19).

Here, we focus on the sociodemographic patterning of respiratory disease in England. Respiratory diseases can often be self-limiting, and therefore may be underreported in national surveillance from traditional data sources such as laboratory reports and hospitalisations. This can lead to bias in the



For peer review only - http://bmjopen.bmj.com/site/about/guidelines.xhtml

reported relationship between healthcare-seeking behaviour for respiratory diseases, and sociodemographic factors. Unlike previous studies, which often focus on traditional data sources (e.g. laboratory reports, hospitalisations), we utilise data sources from telehealth, family doctors and unscheduled care that may provide a more complete reflection of community burden. The data used in this study is defined as non-specific, pre-diagnostic-syndromic data which we use as a proxy for disease (20).

Syndromic data has previously been used to investigate associations between demographics, deprivation and disease. Todkill et al. (21), used syndromic data from family doctors' presentations for allergic rhinitis to investigate sociodemographic associations. Higher rates of allergic rhinitis were observed in females, children and those from more deprived areas. Lusignan et al. (22), used family-doctor coded diagnoses to conduct a large-scale investigation on the impact of age, gender and deprivation on respiratory illnesses in England. Those in the most deprived quintile had a higher probability of presenting with common cold. Nilsson et al. (23), investigated associations between antibiotic prescriptions for penicillin-non-susceptible Streptococcus pneumonia (PNSP) and deprivation in Sweden. Although deprivation was not linked with higher rates of PNSP, higher deprivation was associated with increased rates of antibiotic prescribing.

One issue with the use of syndromic data in such studies is the difficulty in ascertaining whether associations arise from healthcare-seeking behaviours or disease incidence. This situation could be explored by comparison of results to all healthcare-seeking behaviours, but this appears absent in the literature. Furthermore, most studies utilise a single source of syndromic data making it difficult to know the generability of results.

**Aims:**

This study aims to:

1. Explore associations between respiratory-related contacts to three community healthcare services; telehealth, family doctors and unscheduled care, and age, gender and ecological measures of deprivation in England.



For peer review only - http://bmjopen.bmj.com/site/about/guidelines.xhtml

2. Compare these results to all non-respiratory contacts to identify whether associations are specific to respiratory disease.

For peer review only



For peer review only - http://bmjopen.bmj.com/site/about/guidelines.xhtml

## Methods:

### Data Collection

***Syndromic data***

Public Health England (PHE) coordinates a national programme of syndromic surveillance of multiple healthcare services (24). Our study utilises routinely available syndromic data from a telehealth service (NHS111) which operates continuously all year; a family doctor service (General Practitioner In-Hours; GPIH) that operates during weekday working hours, and an out-of-hours family doctor service (General Practitioner Out-of-Hours; GPOOH) (Table 1). These services run as part of the National Health Service (NHS) which is universal and free at the point of delivery.

Syndromic data, obtained from the three surveillance systems coordinated by PHE, comprised of annual counts, between 1 January 2015 and 31 December 2016, of respiratory and non-respiratory contacts. Syndromic indicators classified as respiratory for this study are presented in Table 1 (and described in Appendix 1). Previous studies have demonstrated the association between acute respiratory diseases and these syndromic indicators (25,26). Based on expert knowledge these indicators were chosen to characterise acutely presenting respiratory illnesses such as asthma, and respiratory infections, and to be as comparable as possible between the three healthcare services. Respiratory contacts for each service comprised of the sum of the respiratory indicators listed in Table 1. Non-respiratory counts comprised of the difference between the total number of contacts and the number of respiratory contacts. The total number of contacts was not available through the surveillance system of GPIH, and therefore non-respiratory counts were not available.

Data were obtained at the finest geographical level available; postcode district (PD) (e.g. SW1) for NHS111 and GPOOH, and PHE Centre (e.g. London) for GPIH. In England, there are 2,234 PD with, on average, 25,660 (Range:142-162,266) residents. There are nine PHE Centres with an average of 6,181,375 (Range:2,644,727-9,080,825) residents in England. Count data were subdivided by age group: <1, 1-4, 5-14, 15-44, 45-64, 65-74 and >75 years; by gender (male/female), year (2015/2016); and by geographic location.



For peer review only - http://bmjopen.bmj.com/site/about/guidelines.xhtml

***Demographic data***

PD population, obtained from the 2011 census (27), grouped by age and gender, was used as a denominator in the analysis of NHS111 and GPOOH contacts. GPIH populations were derived from the sum of registered populations at each participating GPIH practice.

***Independent variables***

The Index of Multiple Deprivation (IMD) (28) was used as an area-level measure of deprivation. This index in calculated from seven domains; income, employment, education, health (premature death and poor physical/mental health), crime, barriers to housing and services, and living environment. This index was used, rather than more specific variables (e.g. smoking behaviour) to avoid issues with collinearity. IMD scores were obtained at Lower Layer Super Output Area (LSOA); a weighted mean for each PD was calculated using the proportion of LSOA population in each PD. Weighted means were divided into quintiles from most (1) to least (5) deprived.

**Data Cleaning and Exploration**

NHS111 respiratory contacts from Essex in 2015 & 2016 and Norfolk in 2016 were excluded because syndromic data was unavailable. Syndromic surveillance coverage maps of GPOOH were obtained at the upper tier local authority (UTLA) geographical level, and data were excluded from any UTLA where the PHE surveillance programme received little or no syndromic data.

For all systems, data were excluded if location, age or gender of healthcare seeker was unknown (Table 2). PD that were demarcated for large organisations (e.g. Heathrow Airport), or had less than 200 residents were excluded from analysis as their small populations or unique nature are unlikely to produce reliable estimates. PD that overlap borders with Scotland and Wales were excluded.
For each system, contact rates were mapped for both study years to visualise spatial variation of the data (Appendix 2).



For peer review only - http://bmjopen.bmj.com/site/about/guidelines.xhtml

**Statistical Analysis**

To measure the relationship between the dependent and independent variables generalised linear mixed models (GLMMs) were used for NHS111 and GPOOH, while a generalised linear model (GLM) was used for GPIH. GPIH data was modelled using both GLM and GLMM methods, however the GLM provided a better model fit (Appendix 3).

The variables of interest for analysis of NHS111 and GPOOH data were age, gender, and deprivation. Two-way interactions between age and gender, and age and deprivation were also investigated. A categorical variable for year was included in the model to account for inter-annual variation. Percentage urban area was included to account for differences in healthcare-seeking behaviour or disease risk related to urbanicity. Due to the highly aggregated deprivation and percentage urban data at PHE centre level these variables were not included in the analysis of GPIH data, as non-significant estimates would likely be due to the lack of variation within this aggregated data. Therefore, the variables of interest age and gender and their interactions were investigated in the analysis of GPIH data. To account for population differences at the geographical level, the logarithm of the population plus one was included as a model offset.

The study design accounted for the hierarchical structure of the data for each system by including PD and UTLA as random effects in the NHS111 and GPOOH models. Upper-tier local authority (UTLA) was included as a random effect to account for similar characteristics of neighbouring PD to reduce the effect of spatial autocorrelation, and because UTLA are responsible for services that might influence contact rates (map of UTLA overlaid PD in Appendix 4). Where a PD was located in more than one UTLA, the largest PD area was allocated. PHE Centre was included as a fixed effect in the GPIH model.

We explored Poisson and negative binomial model specifications to account for potential over-dispersion in the data. Over-dispersion was tested by comparing the sum of squared Pearson residuals to the residual degrees of freedom. Models with over-dispersion statistics $<1.5$ were deemed



For peer review only - http://bmjopen.bmj.com/site/about/guidelines.xhtml

acceptable (29). Wald tests were used to determine the overall significance of variables. The algebraic definition of the models is described in Appendix 5.

Model overfitting and the predictive ability of the model was assessed using k-fold cross-validation where the data was split into 10 equal groups (k). Each group was used to train the model k-1 times and test the model once. To assess the model, mean absolute error was used and the results are presented in Appendix 6. Rate ratios (RR) with 95% confidence intervals (CI) were estimated for the main effects: age, gender and deprivation. To allow the visualisation of the main and interaction effects, and comparisons of trends between the contact types and services, the number of contacts to each service were predicted using the models, then standardised, and plotted. The predictions were standardised to a zero mean and unit variance by subtracting the mean of the predictions from each predicted value and then dividing by the standard deviation.

All analyses were conducted in R version 3.5.2 (30) and models were specified using the glmmTMB (31) and MASS (32) packages.

For peer review only



For peer review only - http://bmjopen.bmj.com/site/about/guidelines.xhtml

## Results:

### Data and model selection

Table 2 indicates that relatively few contacts were excluded from analysis due to unknown location, age or gender, with only 3.0% of NHS111 and 11.7% of GPOOH data excluded. GPIH data had no exclusions because patients that used this service have to pre-register and therefore location, age and gender are known. Any data issues observed in the datasets were because of the passive reporting to the surveillance systems and not due to disruption of healthcare services. Table 2 demonstrates that in total 21,242,154 contacts to NHS111 were included in the analysis, of which 8.10% (n = 1,721,034) were respiratory contacts; 6,937,657 GPOOH contacts were included in the analysis of which 22.53% (n = 1,562,883) were respiratory contacts. The different proportions of respiratory contacts between the NHS111 and GPOOH services likely reflect to the different functions of the services, and the severity of illness for which each service would be contacted by patients. Total number of contacts were not available for GPIH, but 10,310,626 respiratory contacts were included in the analysis of GPIH data.

Two model distributions were considered for analysis: Poisson, and negative binomial, with models selected by considering over-dispersion of the data. Negative binomial models handled the over-dispersed data best in all five models (descriptions of model fit in Appendix 7).

Overall, the models performed well with low mean of mean absolute error values (Appendix 6). The fix effects explained a high amount of variation in both NHS111 and GPIH respiratory models, with a marginal $R^2$ of 0.86 and an $R^2$ of 0.99, respectively (Appendix 7). The fixed effects explained less of the variation in the GPOOH respiratory model, with a marginal $R^2$ of 0.30. When the spatial random effects were considered in the NHS111 and GPOOH respiratory models, both models had a conditional $R^2$ of over 0.94. In the GPOOH models the difference observed between marginal and conditional $R^2$ values compared to the NHS1111 models is because less of the variation in the data can be explained by the fixed effects. The difference between the marginal and conditional R-squared in the GPOOH models is likely due to the uncertainty of the underlying study population, with this uncertainty explained by the geographical levels included as random effects (random effects of PD and UTLA).



For peer review only - http://bmjopen.bmj.com/site/about/guidelines.xhtml

**Multivariable Analysis**

Standardised predictions, the number of standard deviations from the mean, of the multivariable analysis for each model are visualised in Figures 1 to 3 alongside overall significance of each model. The table of main effect RR and 95% CI are presented in Appendix 7.

Respiratory contacts in the <1 year age group are significantly higher compared to the reference group (15-44 years) in all three services (Appendix 7), with 37.32 (95%CI:36.10-38.85), 18.66 (95%CI:17.78-19.58), and 6.21 (95%CI:5.96-6.48) times the rate of contacts to NHS111, GPOOH and GPIH, respectively. The comparative differences between respiratory contacts in the <1-year age group and the reference group was highest for NHS111 compared to GPOOH and GPIH. Although contacts are highest in the <1 year age group compared to the reference group in the non-respiratory models, similar RR were observed between the services; with 7.03 (95%CI:6.87-7.20), and 7.64 (95%CI:7.28-8.02) times the rate of non-respiratory contacts to NHS111, and GPOOH. This relationship is visualised in Figure 1a.

Gender has a significant influence on contacts to each service (Appendix 7); visually the trend appears similar between respiratory and non-respiratory contacts and across all services (Figure 1b). There are 1.59 (95%CI:1.56–1.62), 1.73 (95%CI:1.70-1.77), and 1.95 (95%CI:1.87-2.03) times the rate of respiratory contacts regarding females to NHS11, GPOOH and GPIH, respectively.

Deprivation is significant in both NHS111 models (Appendix 7), which found there are 1.75 (95%CI: 1.56-1.95) times the rate of respiratory contacts in the most-deprived areas compared to the least-deprived (IMD quintile 1 vs 5), and 1.81 (95%CI:1.66-1.99) times the rate of non-respiratory contacts. Deprivation was significant in both the respiratory and non-respiratory GPOOH models, with 2.70 (95%CI:1.79-4.08) times the rate of respiratory contacts and 2.70 (95%CI:1.89-3.85) times the rate of non-respiratory contacts in most-deprived areas compared to least-deprived. Similar RR estimates for deprivation were observed in respiratory and non-respiratory NHS111 and GPOOH contacts. This relationship is visualised in Figure 1c, where the similarities between NHS111 and GPOOH, and respiratory and non-respiratory contacts can be observed.



For peer review only - http://bmjopen.bmj.com/site/about/guidelines.xhtml

Age-gender interactions show similar trends across system types and when compared to non-respiratory contacts. Overall, there are more female contacts; but in all models (Figure 2), in the <1 and 1-4 age groups there were more contacts regarding males.

Age-deprivation interactions were investigated for NHS111 and GPOOH. In both respiratory and non-respiratory contacts to NHS111 (Figure 3a), the trends suggest there are more contacts in the most deprived quintiles across all ages. For respiratory contacts, there is a stronger trend with deprivation in the <1, 1-4 and >75-year age groups, and a weaker trend in the remaining age groups. This NHS111 trend is strong across all age groups for non-respiratory contacts, particularly in the age group >75 years. There was a similar linear trend with deprivation and age for respiratory and non-respiratory contacts to GPOOH (Figure 3b), with more contacts in the most-deprived areas in all age groups.

For peer review only



For peer review only - http://bmjopen.bmj.com/site/about/guidelines.xhtml

## Discussion:

### Impact

We present a large-scale analysis of over 13 million respiratory-related contacts to three healthcare services in England. These services deliver healthcare to patients at the community level and may be better indicators of overall disease patterns as opposed to the relatively small proportion that appear in hospitalisation or laboratory-based surveillance. Access to these large syndromic datasets has allowed a comprehensive analysis of the demographics using of each service and the impact deprivation has on healthcare contacts. It has also allowed the interactions between these factors to be explored. By analysing respiratory and non-respiratory contacts, we were able to identify differences and similarities in usage patterns. Analysing NHS111 and GPOOH contacts at PD, a small level of geography, we explored deprivation patterns to give a thorough analysis of social patterning of factors associated with respiratory contacts.

### Main Findings and Comparison to Literature.

In all three services (NHS111, GPOOH and GPIH), there were more respiratory and non-respiratory contacts in females. Previous research indicates that women are more likely to seek healthcare than men, even when female-specific concerns are accounted for (33,34). Although we observed higher respiratory contact rates for in females, males have been observed to have higher death rates due to respiratory disease (9), highlighting possible gender differences in healthcare-seeking behaviour or sex differences in severity of respiratory disease. In terms of age, contacts were highest in <1, followed by the 1-4 and the >75 age groups. These trends were observed across all three services, and fit with previous research (35,36). Although the three services displayed similarities in terms of the age groups more likely to use the service, there were differences in the magnitude of the rate ratios. In comparison to the reference group (15-44 years) children <1 year were 37.3, 18.7 and 6.2 times more likely to contact NHS111, GPOOH and GPIH, respectively, due to respiratory disease. These differences could reflect the routes to access to the different services (Table 1), and the severe and sudden nature of the respiratory illness in very young children requiring urgent health advice.



For peer review only - http://bmjopen.bmj.com/site/about/guidelines.xhtml

When interactions between age and gender were explored, we found that more contacts were regarding males in the two youngest age groups for both respiratory diseases; this effect was observed in all services. Previous studies have reported that male children have higher rates of respiratory illnesses (17,35,37). Lusignan et al. (37), observed a higher incidence of family doctors contacts in males <15 years due to LRTI compared to females of the same age, with the largest gender difference in the youngest age group (<1 year). This study also noted a higher contact rate in males <15 years due to asthma. The reasons for which are unclear, but immunologic, genetic and biological differences are thought to increase risk, suggesting that the excess in male children is due to genuine predisposition to respiratory illness rather than sociological factors (16). This excess in male children was also observed in our data for non-respiratory contacts. The reasons for this excess of non-respiratory contacts in male children is unclear. These findings suggest that there are similar drivers to contacts in male children presenting for all illness types rather than with just respiratory diseases, whether this be due to differences in healthcare-seeking behaviours or disease aetiology. Earp et al. (2019) observed that adults perceived that male children (aged 5-years) experienced more pain compared to females of the same age, despite the same clinical circumstances and identical pain behaviour (38). The Infectious Intestinal Disease (IID) Study observed a higher rate of IID in the community in females <1-year compared to males, but a higher rate of contacts to family doctors of males compared to females of the same age (39). This observation suggests that either males were more likely to have cases of IID, which required medical intervention, or healthcare was more likely to be sought for males compared to females of the same age. There could be gender differences in the perception of ill health in young children from parental caregivers, which could influence healthcare-seeking behaviour, although this requires more research.

Deprivation was significant in both the NHS111 and GPOOH models, with a greater risk of respiratory and non-respiratory contacts in those from more deprived areas. Similar results were found when looking at gastrointestinal illness contacts to NHS111 and deprivation, with more contacts in the most deprived areas (40). Kelly et al. (41), observed higher attendances from those from the most deprived areas to GPOOH services. The findings observed in this study are similar to those described in the literature from more acute healthcare settings, with previous studies linking



For peer review only - http://bmjopen.bmj.com/site/about/guidelines.xhtml

increased deprivation with increased rates of hospitalisations and death due to respiratory infection (9,42,43).

One of the relatively unique elements of our work was the exploration of the interactions between deprivation and age. For NHS111, when stratified by age, deprivation appears to have a greater impact on non-respiratory contacts than on respiratory contacts. The exception is in the youngest age bracket, where deprivation has a similarly high impact on both respiratory and non-respiratory contacts, with more contacts in most-deprived areas. A similar pattern emerged for GPOOH where there was more contact in the most derived areas; however, this trend was similar across age groups and in respiratory and non-respiratory contacts.

It is important to consider how these results can be generalised outside of England. It is difficult to make direct comparisons to other countries due to differences in healthcare systems, public health surveillance infrastructure, population demographics and deprivation levels. With the rise internationally in use of syndromic and non-traditional forms of surveillance (such as those used in this study), we feel it is important for other counties who use these forms of surveillance to undertake similar research.

**Limitations**

Although the surveillance of these three services allows us to observe a large number of healthcare contacts, these are working surveillance datasets from real-time surveillance system, with periods where data is not received. The large numbers included in the study meant that socioeconomic and demographic patterns could still be observed, and omissions are only likely to bias estimates if correlated with independent variables. Coverage issues will increase the uncertainty of our estimates, which is particularly evident in the wide confidence intervals observed in the GPOOH results.

The analysis presented here is exploring indicators of disease as opposed to actual disease. Therefore, the analyses are reliant on accurate classifications of disease indicators. NHS111, GPIH and GPOOH services have different symptom coding systems, and although we tried to choose indicators which would allow comparisons, the definition of respiratory and non-respiratory may differ between services. The deprivation measures used were composites of multiple factors that contribute to

For peer review only



For peer review only - http://bmjopen.bmj.com/site/about/guidelines.xhtml

deprivation and were ecological in nature, are subject to the ecological fallacy whereby the associations found at the area level may not hold at the individual level. Data obtained from the surveillance systems did not contain information on co-morbidities or ethnicity, therefore these factors could not be accounted for in the analysis. Data from each surveillance system was available in an aggregated format, and hence it was not possible to identify multiple contacts by the same patient to the services during the study period.

**Conclusion**

This large-scale study highlights the effects of age, gender and deprivation on healthcare-seeking behaviours for respiratory diseases in the community, with more healthcare contacts from females, the young and old and those from more deprived areas. Similar patterns were observed across the three services, which were in agreement with the literature. Comparable results were also observed between respiratory and non-respiratory contacts suggesting that even when a wider spectrum of disease is explored, demographic and socioeconomic factors may be the strongest influencers of healthcare-seeking behaviours. When broken down by age there were more contacts regarding male children <5 years compared to females. This trend was observed in both respiratory and non-respiratory contacts indicating there are more healthcare-seeking behaviours in male children across a range of disease types. These findings could be influenced by sociological factors as well as disease aetiology and requires further research. Further research is required to understand the role that age, gender and deprivation has on healthcare-seeking behaviours.

For peer review only



For peer review only - http://bmjopen.bmj.com/site/about/guidelines.xhtml

Table 1 Indicators for respiratory diseases for each syndromic surveillance system, 1st January 2015 to 31st December 2016.

| Healthcare Service | Healthcare Service Provided | Coding System for Healthcare Service | Contact Type | Routine syndromic indicator included for this study | Number of Contacts used in study |
|---|---|---|---|---|---|
| NHS111 | NHS111 is a free non-emergency medical helpline. It operates 24/7 and is staffed by trained call handlers. A clinical decision support system is used to structure the response to the call, with the call disposal ranging from advice about self-care to dispatch of an emergency ambulance. | NHS Pathways (44) | Acute respiratory consultations | "Cold/flu", "cough" and "difficulty breathing" | 1,721,034 |
| | | | Total number of consultations | All contacts | 21,242,154 |
| | | | Non-acute respiratory | All non-acute respiratory contacts (All contacts – acute respiratory contacts) | 19,521,120 |
| GPIH | GPIH are primary care services that provide free scheduled day-to-day healthcare in England. General practitioners (GPs) treat all common medical conditions and depending on the condition will refer patients to hospitals and other medical services for urgent and specialist treatment. | Read codes v2 (hierarchical) and v3 (non-hierarchical). Full description in Robinson et al. (45) | Acute respiratory consultations | "Upper and lower respiratory tract infections" and "asthma" | 10,310,626 |
| | | | Total number of consultations | Not available | Not available |
| | | | Non-respiratory | Not available | Not available |
| GPOOH | GPOOH services provide free access to primary healthcare when GPIH services are closed, which is typically weekdays 6:30pm – 8:00am, weekends and bank holidays. | Read codes (45) | Acute respiratory consultations | "Acute respiratory infection" "difficulty breathing/wheeze/asthma" | 1,562,883 |
| | | | Total number of consultations | All contacts | 8,500,540 |
| | | | Non-acute respiratory | All non-acute respiratory contacts (All contacts – acute respiratory contacts) | 6,937,657 |

NHS – National Health Service, GPIH – general practitioner in hours; GPOOH – general practitioner out of hours



For peer review only - http://bmjopen.bmj.com/site/about/guidelines.xhtml

Table 2. Number of contacts excluded from study by reason for exclusion.

| | **NHS111 (% of total)** | **GPOOH (% of total)** |
|---|---|---|
| Total number provided | 21,905,099 | 9,623,939 |
| **Reason for exclusion** | | |
| No valid postcode provided/Not in England | 613,495 (2.9) | 92,815 (9.6) |
| No gender given | 9,536 (0.04) | 12,312 (0.13) |
| No age given | - | 1,601 (0.017) |
| Postcode District with < 200 population | 44 (0.0002) | 12 (0.0001) |
| Overlapping borders with Scotland or Wales | 17,072 (0.08) | 3,795 (0.04) |
| Large area users/City centres | 1,517 (0.007) | 2,379 (0.03) |
| Poor coverage/Data Issues | 21,281 (0.1) | 1,010,485 (10.50) |
| **Total excluded** | **662,945 (3.02)** | **1,123,399 (11.67)** |

[+]GPIH had no exclusions

For peer review only

**Acknowledgements**

The authors acknowledge technical support from Paul Loveridge, Ana Soriano, and Helen Hughes (PHE Real-time Syndromic Surveillance Team) and support from: NHS 111 and NHS Digital for their assistance and support with the NHS 111 system; OOH providers submitting data to the GPOOH and Advanced; TPP and participating SystmOne practices and University of Oxford, ClinRisk, EMIS and EMIS practices submitting data to the QSurveillance database.

KEM, FCG, PRH, AJE, GES and IRL are supported by the National Institute for Health Research Health Protection Research Unit (NIHR HPRU) in Emergency Preparedness and Response at King's College London in partnership with PHE. The views expressed are those of the author(s) and not necessarily those of the NHS, the NIHR, the Department of Health or Public Health England.

**Data Statement**

Sharing: No additional data available.

**Ethics**

Ethical approval for this work was not required. This study was undertaken as part of the national surveillance functions of Public Health England (PHE). The anonymised, aggregated health data used in this study, were routinely collected as part of the public health function of PHE.

**Funding**

KEM is supported by the National Institute for Health Research and University of East Anglia FCG, PRH, AJE, GES and IRL are supported by the NIHR HPRU in Emergency Preparedness and Response.

**Patient and Public Involvement**

There was no patient or public involvement in the design of this study.

**Conflicts of Interest**

None Declared

For peer review only

## References


1. Troeger C, Blacker B, Khalil IA, Rao PC, Cao J, Zimsen SRM, et al. Estimates of the global, regional, and national morbidity, mortality, and aetiologies of lower respiratory infections in 195 countries, 1990–2016: a systematic analysis for the Global Burden of Disease Study 2016. The Lancet Infectious Diseases. 2018 Nov 1;18(11):1191–210.

2. Hay SI, Abajobir AA, Abate KH, Abbafati C, Abbas KM, Abd-Allah F, et al. Global, regional, and national disability-adjusted life-years (DALYs) for 333 diseases and injuries and healthy life expectancy (HALE) for 195 countries and territories, 1990–2016: a systematic analysis for the Global Burden of Disease Study 2016. The Lancet. 2017 Sep 16;390(10100):1260–344.

3. Soriano JB, Abajobir AA, Abate KH, Abera SF, Agrawal A, Ahmed MB, et al. Global, regional, and national deaths, prevalence, disability-adjusted life years, and years lived with disability for chronic obstructive pulmonary disease and asthma, 1990–2015: a systematic analysis for the Global Burden of Disease Study 2015. The Lancet Respiratory Medicine. 2017 Sep 1;5(9):691–706.

4. Global Burden of Disease Collaborative Network. Global Burden of Disease Study 2010 (GBD 2010) Results by Risk Factor 1990-2010. Institute for Health Metrics and Evaluation (IHME), Seattle, USA; 2012.

5. Murray CJ, Richards MA, Newton JN, Fenton KA, Anderson HR, Atkinson C, et al. UK health performance: findings of the Global Burden of Disease Study 2010. The Lancet. 2013 Mar 23;381(9871):997–1020.

6. Mukherjee M, Stoddart A, Gupta RP, Nwaru BI, Farr A, Heaven M, et al. The epidemiology, healthcare and societal burden and costs of asthma in the UK and its member nations: analyses of standalone and linked national databases. BMC Medicine. 2016 Aug 29;14(1):113.

7. Trueman D, Woodcock F, Hancock E. Estimating the economic burden of respiratory illness in the UK [Internet]. British Lung Foundation; 2017. Available from: https://www.probonoeconomics.com/sites/default/files/files/British%20Lung%20Foundation%20full%20report%2015032017_0.pdf

8. Sallakh MAA, Rodgers SE, Lyons RA, Sheikh A, Davies GA. Socioeconomic deprivation and inequalities in asthma care in Wales. The Lancet. 2017 Nov 1;390:S19.

9. Jordan R, Verlander N, Olowokure B, Hawker JI. Age, sex, material deprivation and respiratory mortality. Respiratory Medicine. 2006 Jul;100(7):1282–5.

10. Riaz SP, Horton M, Kang J, Mak V, Lüchtenborg M, Møller H. Lung cancer incidence and survival in England: an analysis by socioeconomic deprivation and urbanization. Journal of Thoracic Oncology. 2011 Dec;6(12):2005–10.

11. Roberts SE, Button LA, Hopkin JM, Goldacre MJ, Lyons RA, Rodgers SE, et al. Influence of social deprivation and air pollutants on serious asthma. European Respiratory Journal. 2012 Sep 1;40(3):785–8.

12. Newton JN, Briggs ADM, Murray CJL, Dicker D, Foreman KJ, Wang H, et al. Changes in health in England, with analysis by English regions and areas of deprivation, 1990–2013: a systematic analysis for the Global Burden of Disease Study 2013. The Lancet. 2015 Dec 5;386(10010):2257–74.


For peer review only

13. Sofianopoulou E, Rushton SP, Diggle PJ, Pless-Mulloli T. Association between respiratory prescribing, air pollution and deprivation, in primary health care. Journal of Public Health (Oxf). 2013 Dec;35(4):502–9.

14. Gibson M, Petticrew M, Bambra C, Sowden AJ, Wright KE, Whitehead M. Housing and health inequalities: a synthesis of systematic reviews of interventions aimed at different pathways linking housing and health. Health Place. 2011 Jan;17(1):175–84.

15. Falagas ME, Mourtzoukou EG, Vardakas KZ. Sex differences in the incidence and severity of respiratory tract infections. Respiratory Medicine. 2007 Sep;101(9):1845–63.

16. Liptzin DR, Landau LI, Taussig LM. Sex and the lung: Observations, hypotheses, and future directions. Pediatric Pulmonology. 2015 Dec;50(12):1159–69.

17. Vink NM, Postma DS, Schouten JP, Rosmalen JGM, Boezen HM. Gender differences in asthma development and remission during transition through puberty: the TRacking Adolescents' Individual Lives Survey (TRAILS) study. The Journal of Allergy and Clinical Immunology. 2010 Sep;126(3):498-504.e1-6.

18. Almqvist C, Worm M, Leynaert B, working group of GA2LEN WP 2.5 Gender. Impact of gender on asthma in childhood and adolescence: a GA2LEN review. Allergy. 2008 Jan;63(1):47–57.

19. Bundle N, Verlander NQ, Morbey R, Edeghere O, Balasegaram S, Lusignan S de, et al. Monitoring epidemiological trends in back to school asthma among preschool and school-aged children using real-time syndromic surveillance in England, 2012–2016. Journal of Epidemiology and Community Health. 2019 Sep 1;73(9):825–31.

20. Triple S Project. Assessment of syndromic surveillance in Europe. The Lancet. 2011 Dec 2;378(9806):1833–4.

21. Todkill D, Hughes HE, Elliot AJ, Morbey RA, Edeghere O, Harcourt S, et al. An Observational Study Using English Syndromic Surveillance Data Collected During the 2012 London Olympics – What did Syndromic Surveillance Show and What Can We Learn for Future Mass-gathering Events? Prehospital and Disaster Medicine. 2016 Jan;1–7.

22. Lusignan S de, Correa A, Pathirannehelage S, Byford R, Yonova I, Elliot AJ, et al. RCGP Research and Surveillance Centre Annual Report 2014–2015: disparities in presentations to primary care. British Journal of General Practice. 2017 Jan 1;67(654):e29–40.

23. Nilsson P, Laurell MH. Impact of socioeconomic factors and antibiotic prescribing on penicillin- non-susceptible Streptococcus pneumoniae in the city of Malmö. Scandinavian Journal of Infectious Diseases. 2005 Jan;37(6–7):436–441.

24. Public Health England (PHE). Syndromic surveillance: systems and analyses [Internet]. 2020 [cited 2020 Jun 29]. Available from: https://www.gov.uk/government/collections/syndromic-surveillance-systems-and-analyses

25. Morbey RA, Harcourt S, Pebody R, Zambon M, Hutchison J, Rutter J, et al. The burden of seasonal respiratory infections on a national telehealth service in England. Epidemiology and Infection. 2017 Jul;145(9):1922–32.

26. Morbey RA, Elliot AJ, Harcourt S, Smith S, de Lusignan S, Pebody R, et al. Estimating the burden on general practitioner services in England from increases in respiratory disease

For peer review only

associated with seasonal respiratory pathogen activity. Epidemiology and Infection. 2018 Aug;146(11):1389–96.

27. Office for National Statistics, National Records of Scotland, Northern Ireland Statistics and Research Agency. 2011 Census aggregate data. 2017;UK Data Service (Edition: February 2017).

28. Smith T, Noble M, Noble S, Wright G, McLennan D, Plunkett E. English indices of deprivation 2015: technical report. London: Department for Communities and Local Government; 2015 Sep.

29. Payne EH, Gebregziabher M, Hardin JW, Ramakrishnan V, Egede LE. An empirical approach to determine a threshold for assessing overdispersion in Poisson and negative binomial models for count data. Communications in Statistics: Simulation and Computation. 2018 Jul;47(6):1722–1738.

30. R Core Team. R: A language and environment for statistical computing. R Foundation for Statistical Computing [Internet]. Vienna, Austria; 2017. Available from: https://www.R-project.org/

31. Brooks ME, Kristensen K, van Benthem KJ, Magnusson A, Berg CW, Nielsen A, et al. glmmTMB Balances Speed and Flexibility Among Packages for Zero-inflated Generalized Linear Mixed Modeling. The R Journal. 2017;9(2):378–400.

32. Venables WN, Ripley BD. Modern Applied Statistics with S [Internet]. Fourth. New York: Springer; 2002. Available from: http://www.stats.ox.ac.uk/pub/MASS4

33. Manierre MJ. Gaps in knowledge: tracking and explaining gender differences in health information seeking. Social Science & Medicine. 2015 Mar;128:151–8.

34. Thompson AE, Anisimowicz Y, Miedema B, Hogg W, Wodchis WP, Aubrey-Bassler K. The influence of gender and other patient characteristics on health care-seeking behaviour: a QUALICOPC study. BMC Family Practice. 2016 Mar 31;17(1):38.

35. Liptzin DR, Landau LI, Taussig LM. Sex and the lung: Observations, hypotheses, and future directions. Pediatric Pulmonology. 2015 Dec 1;50(12):1159–69.

36. Salisbury C, Trivella M, Bruster S. Demand for and supply of out of hours care from general practitioners in England and Scotland: observational study based on routinely collected data. BMJ. 2000 Mar;320(7235):618–621.

37. de Lusignan S, Correa A, Pebody R, Yonova I, Smith G, Byford R, et al. Incidence of Lower Respiratory Tract Infections and Atopic Conditions in Boys and Young Male Adults: Royal College of General Practitioners Research and Surveillance Centre Annual Report 2015-2016. JMIR Public Health Surveill. 2018 Apr 30;4(2):e49.

38. Earp BD, Monrad JT, LaFrance M, Bargh JA, Cohen LL, Richeson JA. Featured Article: Gender Bias in Pediatric Pain Assessment. Journal of Pediatric Psychology. 2019 Jan 4;44(4):403–14.

39. Food Standards Agency. A Report of the Study of Infectious Intestinal Disease in England. London HMSO; 2000. Available from :https://www.food.gov.uk/sites/default/files/media/document/iid1_study_final_report.pdf

40. Adams NL, Rose TC, Elliot AJ, Smith G, Morbey R, Loveridge P, et al. Social patterning of telephone health-advice for diarrhoea and vomiting: analysis of 24 million telehealth calls in

For peer review only

England. Journal of Infection. 2019;78(2):95-100; Available from: http://www.sciencedirect.com/science/article/pii/S0163445318302810

41. Kelly JS, Piercy H, Ibbotson R, Davis SVF. Who attends out-of-hours general practice appointments? Analysis of a patient cohort accessing new out-of-hours units. BMJ Open. 2018 Jun;8(6):e020308.

42. Hawker JI, Olowokure B, Sufi F, Weinberg J, Gill N, Wilson RC. Social deprivation and hospital admission for respiratory infection: an ecological study. Respiratory Medicine. 2003 Nov;97(11):1219–24.

43. Geyer S, Peter R, Siegrist J. Socioeconomic differences in children's and adolescents' hospital admissions in Germany: a report based on health insurance data on selected diagnostic categories. Journal of Epidemiology and Community Health. 2002 Feb;56(2):109–14.

44. NHS Digital. NHS Pathways [Internet]. 2020 [cited 2020 Jun 28]. Available from: https://digital.nhs.uk/services/nhs-pathways

45. Robinson D, Schulz E, Brown P, Price C. Updating the Read Codes: user-interactive maintenance of a dynamic clinical vocabulary. Journal of the American Medical Informatics Association. 1997 Dec;4(6):465–72.

**Figure Captions**

Figure 1. Standard deviations from the mean of main effects predictions from the multivariable analysis of respiratory contacts to NHS111, General Practitioner-out-of-hours (GPOOH) and General Practitioner-in-hours (GPIH): Each plot describes the standard deviations of A) age group, B) gender and C) deprivation. All models had an overall significance ≤0.001.

Figure 2. Standard deviations from the mean of age gender interactions for respiratory and non-respiratory contacts by each system: A) NHS111, B) General Practitioner-in-hours (GPIH) and C) General Practitioner-out-of-hours (GPOOH). All models had an overall significance ≤0.001.

Figure 3. Standard deviations from the mean of age deprivation interactions for respiratory and non-respiratory contacts by each system: A) NHS111 and B) General Practitioner-out-of-hours (GPOOH). NHS111 respiratory, GPOOH respiratory and GPOOH non-respiratory models had an overall significance ≤0.001. NHS111 non-respiratory model had an overall significance of ≤0.01

For peer review only

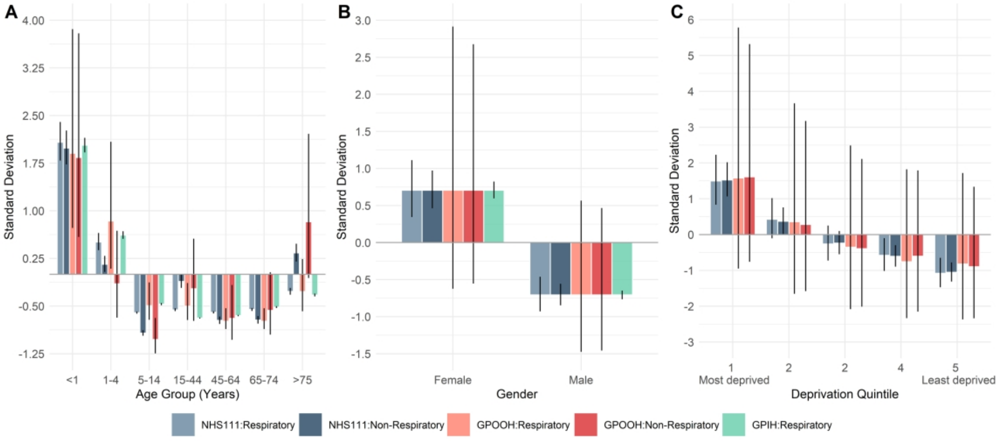


. Standard deviations from the mean of main effects predictions from the multivariable analysis of respiratory contacts to NHS111, General Practitioner-out-of-hours (GPOOH) and General Practitioner-in-hours (GPIH): Each plot describes the standard deviations of A) age group, B) gender and C) deprivation. All models had an overall significance ≤0.001.

342x152mm (300 x 300 DPI)

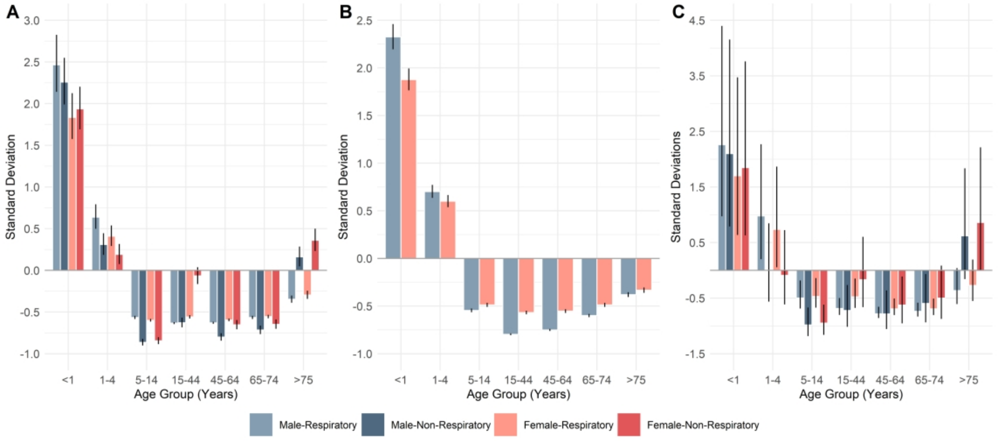


Standard deviations from the mean of age gender interactions for respiratory and non-respiratory contacts by each system: A) NHS111, B) General Practitioner-in-hours (GPIH) and C) General Practitioner-out-of-hours (GPOOH). All models had an overall significance ≤0.001.

342x152mm (300 x 300 DPI)

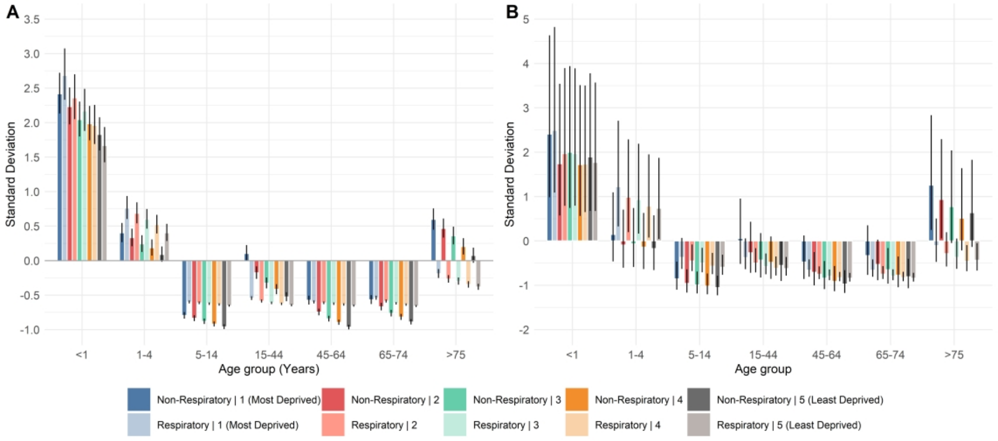


Standard deviations from the mean of age deprivation interactions for respiratory and non-respiratory contacts by each system: A) NHS111 and B) General Practitioner-out-of-hours (GPOOH). NHS111 respiratory, GPOOH respiratory and GPOOH non-respiratory models had an overall significance ≤0.001. NHS111 non-respiratory model had an overall significance of ≤0.01

Supplementary Material

Appendix 1. Descriptions of Syndromic Indicators used in study

| Healthcare Service | Syndromic Indicator | Description |
|---|---|---|
| NHS111 | Difficulty breathing | Patients calling NHS111 and reporting symptoms of breathing problems, breathlessness or wheeze<br>This syndromic indicator excludes those callers reporting symptoms of difficulty breathing which the call handler assesses as being of immediate threat to life for which an emergency ambulance is required. These patients would not routinely continue with telephone triage and therefore would not have a Pathway selected. |
| | Cough | Patients calling NHS111 and reporting symptoms of cough |
| | Cold/flu | Patients calling NHS111 and reporting symptoms of colds and/or influenza |
| GPIH | Upper respiratory tract infection (URTI) | Codes suggestive of an acute bacterial or viral infection mainly affecting the upper respiratory tract (includes colds/flu, sinusitis, throat infections, ear infections). |
| | Lower respiratory tract infection (LRTI) | Codes suggestive of an acute bacterial or viral infection mainly affecting the lower respiratory tract including pneumonia, bronchitis, bronchiolitis, pleurisy and complications. Includes codes for the pneumonia indicator. |
| GPOOH | Asthma | Diagnoses of acutely presenting or severe asthma including asthma attack, not including routine consultations (where possible to distinguish and not including prescription links) |
| | Acute respiratory infection (ARI) | Acute respiratory infection - includes all codes indicative of an acute respiratory infection eg. acute sinusitis, viral pneumonia, influenza and pleurisy |
| | Difficulty breathing/wheeze/asthma | Difficulty breathing/wheeze/asthma - includes codes indicative of asthma, wheeze and difficulty breathing. Includes eg. dyspnoea, bronchial breathing, expiratory wheeze and stridor. |

For peer review only

Appendix 2. Spatial variation of contacts to three health services in England between 1st January 2015 & 31st December 2016

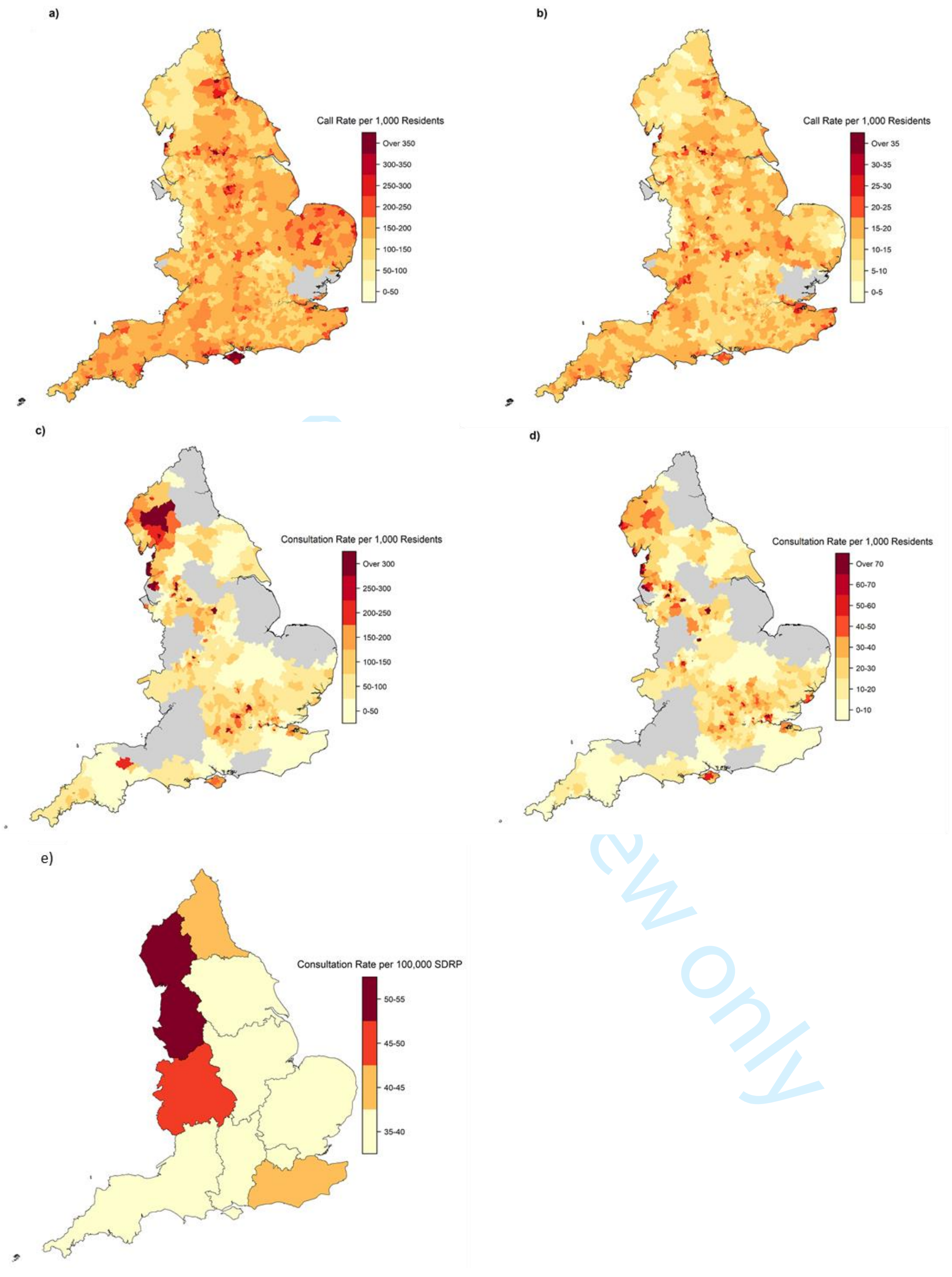


a) All non-respiratory calls to NHS111 at PD,
b) Respiratory calls to NHS111 at PD,
c) All non-respiratory consultations to GPOOH services at PD,
d) Respiratory consultations to GPOOH services at PD,
e) Respiratory calls to GPIH services PHE centre. SDPR = Sum of daily registered population
The grey areas indicate the PD that were excluded for the duration of the study period.

Appendix 3. Comparison between GLM and GLMM results of respiratory calls to GPIH (ns = not significant (overall effect only), * = ≤0.05, ** = ≤0.01, *** = ≤0.001).

| | GLM | | GLMM | |
|---|---|---|---|---|
| Variable | **Rate** Ratio | 95% CI | **Rate** Ratio | 95% CI |
| Intercept | 142.693 | 0.000 –1.455e+16 | 142.264 | 0.000-2.619e+16 |
| Main Effects | | | | |
| Age | | *** | | *** |
| Under 1 year | 6.213 | 5.955 – 6.482*** | 6.213 | 5.950-6.487*** |
| 1-4 years | 3.492 | 3.348 – 3.643*** | 3.492 | 3.450-3.645*** |
| 5-14 years | 1.172 | 1.123 – 1.223*** | 1.172 | 1.123 - 1.223*** |
| 15-44 years | ref | ref | ref | ref |
| 45-64 years | 1.032 | 0.989 – 1.076 | 1.032 | 0.988 - 1.077 |
| 65-74 years | 1.170 | 1.122 – 1.221*** | 1.170 | 1.121 - 1.222*** |
| 75 years and above | 1.500 | 1.438 – 1.564*** | 1.500 | 1.436 - 1.566*** |
| Gender | | | | *** |
| Female | ref | ref | ref | ref |
| Male | 0.513 | 0.492 – 0.535*** | 0.513 | 0.492-0.536*** |
| PHE Centre | - | *** | - | - |
| Year | - | ns | - | ns |
| Interaction Effects | | | | |
| Age:Gender | - | *** | - | *** |
| AIC | | 5083.492 | | 5117.537 |
| Deviance Explained | | 0.993 | | 0.992 |
| Dispersion Statistic | | 1.100 | | 1.0332 |

For peer review only

Appendix 4. Map of upper teir local authorties over-layed postcode dictricts

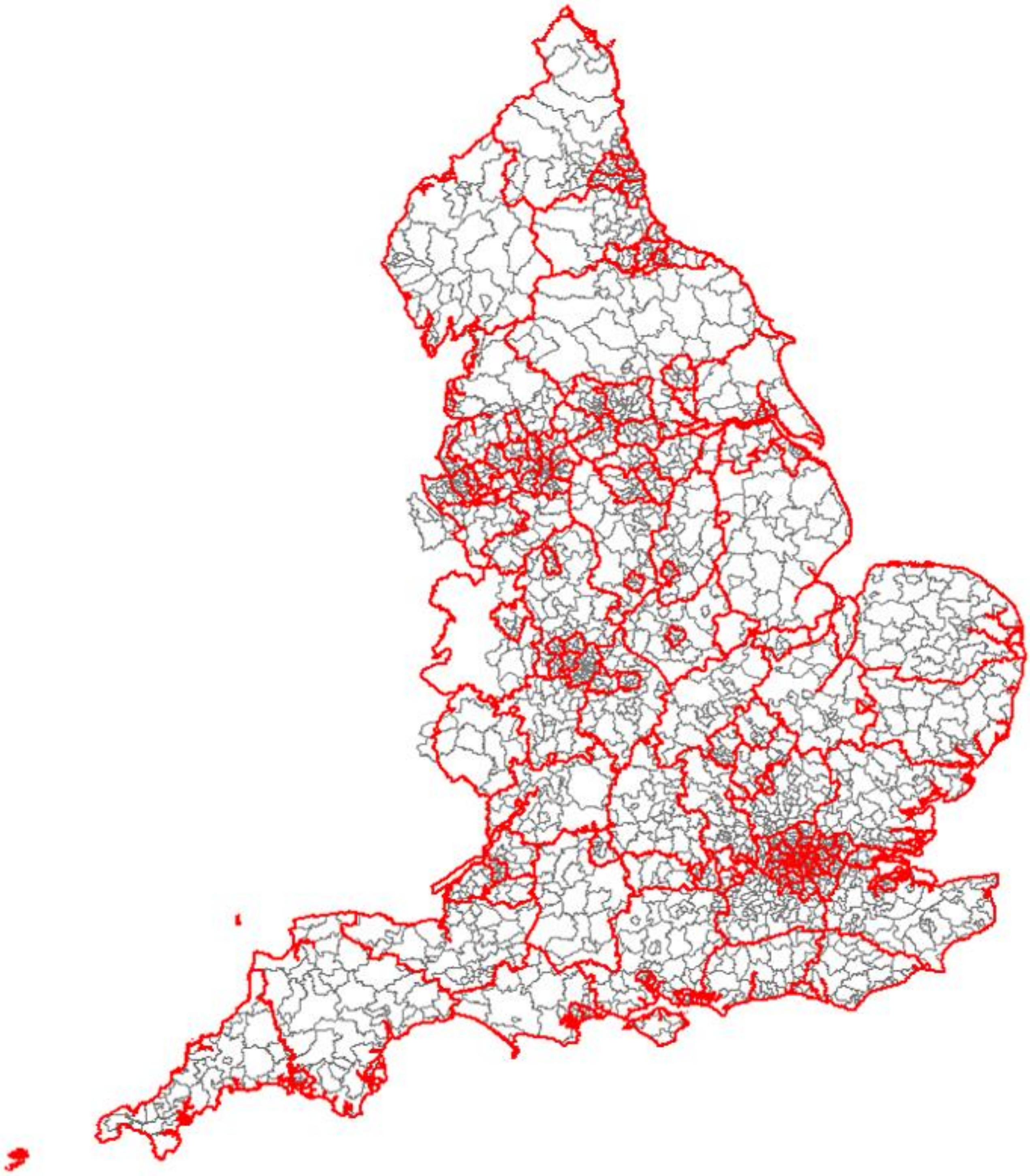

*Upper-tier local authorities (red) overlapped over PD (grey)*

Appendix 5. Algebraic definition of the models

The general algebraic definition of the models is given by:

$$Ya,g,i,y \mid \mu a,g,i,y,\varphi \sim NegBin(\mu a,g,i,y,\varphi),$$

Where $\mu_{a,g,i,y}$ is the is the number of age *a* and gender *g* specific contacts at each geographical location (PHE centre or postcode district) *i* at year *y*, and $\varphi > 0$ is the negative binomial dispersion parameter. The expected number of cases for GPIH services is modelled as:

$$\log(\mu_{a,g,i,y}) = \alpha + \log(P_{a,g,i,y}) + \beta a + \gamma_g + \delta_y + \epsilon_i + \beta a \cdot \gamma_g$$

Where $\alpha$ corresponds to the intercept; $\log(P_{a,g,i,y})$ denotes the logarithm of the population *P* at risk for PHE centre *i*, age *a*, gender *g* and year *y* included as an offset to adjust counts by population; *a* denotes a categorical variable age with coefficient $\beta$; *g* denotes a categorical variable gender with coefficient $\gamma$; *y* denotes a categorical variable for each year with coefficient $\delta$; *i* denotes a categorical variable PHE centre with coefficient $\epsilon$. The interaction between age and gender is denoted by $\beta a \cdot \gamma_g$.

The expected number of cases for NHS111 and GPOOH services is modelled as:

$$\log(\mu a,g,i,y) = \alpha + \log(Pa,g,i,y) + \beta a + \gamma g + \delta y + \zeta d + \eta p + \beta a\cdot\gamma g + \beta a\cdot\zeta d + ui + vl$$

Where $\alpha$ corresponds to the intercept; $\log(P_{a,g,i,y})$ denotes the logarithm of the population *P* at risk for postcode district *i*, age *a*, gender *g* and year *y* included as an offset to adjust counts by population at risk; *a* denotes a categorical variable age with coefficient $\beta$; *g* denotes a categorical variable gender with coefficient $\gamma$; *y* denotes a categorical variable for year with coefficient $\delta$; *d* denotes a categorical variable for deprivation with coefficient $\zeta$; *p* denotes a continuous variable to represent the percentage of postcode district that is classified as urban with coefficient $\eta$. $\beta a\cdot\gamma_g$ denotes the interaction between age and gender, and $\beta a\cdot\zeta_d$ denotes the interaction between age and deprivation. Unstructured random effects of postcode district ($u_i$) and upper tier local authority ($v_l$) were included to account for unknown confounding factors, such as temperature, the hierarchical structure of the data and spatial dependency.

Appendix 6. Cross validation of final model to assess model overfitting.

| | NSH111 | | GPOOH | | GPIH |
|---|---|---|---|---|---|
| | Respiratory | Non-Respiratory | Respiratory | Non-Respiratory | Respiratory |
| Max number of contacts per location | 391 | 17,842 | 2,108 | 10,175 | 200,304 |
| Min number of contacts per location | 0 | 0 | 0 | 0 | 4,274 |
| Mean number of contacts per location | 30.9 | 350.3 | 39.2 | 173.9 | 36,823.7 |
| Mean of the mean absolute error | 7.02 | 54.6 | 9.5 | 40.5 | 2,533.7 |

For peer review only

Appendix 7. Final Model multivariable regression analysis of respiratory and non-respiratory contacts to three healthcare services. Full results and overall significance presented for main effects, and overall significance for interaction terms (ns = not significant, * = ≤0.05, ** = ≤0.01, *** = ≤0.001, x = variable not modelled, - = reference level).

| | **NHS 111** | | | | **GPOOH** | | | | **GPIH** | |
|---|---|---|---|---|---|---|---|---|---|---|
| | **Respiratory Calls** | | **Non-respiratory Calls** | | **Respiratory Calls** | | **Non-respiratory Calls** | | **Respiratory Calls (PHE Centre** | |
| **Variable** | **Rate Ratio** | **95% CI** | **Rate Ratio** | **95% CI** | **Rate Ratio** | **95% CI** | **Rate Ratio** | **95% CI** | **Rate Ratio** | **95% CI** |
| Intercept | 0.005 | 0.004-0.005 | 0.106 | 0.099-0.115 | 0.002 | 0.001-0.003*** | 0.015 | 0.009-0.023*** | 142.693 | 0.000 – 1.455e+16 |
| **Main Effects** | | | | | | | | | | |
| %Urban | 1.002 | 1.002-1.003*** | 1.001 | 1.001-1.002*** | 1.004 | 1.003-1.006*** | 1.004 | 1.003-1.006*** | x | x |
| Age | – | *** | – | *** | – | *** | – | *** | – | *** |
| *Under 1 year* | 37.324 | 36.104-38.850*** | 7.031 | 6.869-7.197*** | 18.661 | 17.783-19.581*** | 7.640 | 7.281-8.018*** | 6.213 | 5.955 – 6.482*** |
| *1 – 4 years* | 16.683 | 16.177-17.206*** | 3.220 | 3.149-3.292*** | 9.822 | 9.400-10.263*** | 2.782 | 2.656-2.913*** | 3.492 | 3.348 – 3.643*** |
| *5 – 14 years* | 1.983 | 1.915-2.054*** | 0.871 | 0.851-0.891*** | 1.879 | 1.794-1.966*** | 0.791 | 0.755-0.829*** | 1.172 | 1.123 – 1.223*** |
| *15-44 years* | ref | ref | ref | ref | ref | ref | ref | ref | ref | ref |
| *45-64 years* | 0.854 | 0.826-0.883*** | 0.670 | 0.656-0.685*** | 0.515 | 0.492-0.540*** | 0.795 | 0.761-0.831*** | 1.032 | 0.989 – 1.076 |
| *65-74 years* | 1.372 | 1.324-1.423*** | 0.912 | 0.892-0.932*** | 0.674 | 0.640-0.709*** | 1.247 | 1.192-1.304*** | 1.170 | 1.122 – 1.221*** |
| *75 years and over* | 3.999 | 3.872-4.130*** | 2.673 | 2.616-2.732*** | 2.500 | 2.388-2.617*** | 4.029 | 3.855-4.211*** | 1.500 | 1.438 – 1.564*** |
| Deprivation Quintile | – | *** | – | *** | – | *** | – | *** | – | – |
| *(Most Deprived) 1* | 1.745 | 1.561-1.949*** | 1.814 | 1.658-1.985*** | 2.704 | 1.792-4.081*** | 2.700 | 1.892-3.853*** | x | x |
| *2* | 1.382 | 1.291-1.480*** | 1.387 | 1.313-1.466*** | 1.654 | 1.326-2.062*** | 1.659 | 1.374-2.003*** | x | x |
| *3* | 1.172 | 1.104-1.245*** | 1.179 | 1.124-1.236*** | 1.318 | 1.089-1.596** | 1.231 | 1.045-1.451* | x | x |

| | | | | | | | | | | |
|---|---|---|---|---|---|---|---|---|---|---|
| *4* | 1.075 | 1.019-1.133** | 1.085 | 1.040-1.132*** | 1.153 | 0.986-1.153 | 1.200 | 1.050-1.372** | x | x |
| *(Least Deprived) 5* | ref | ref | ref | ref | ref | ref | ref | ref | x | x |
| Gender | – | *** | | *** | – | *** | – | *** | – | – |
| *Male* | ref | ref | ref | ref | ref | ref | ref | ref | ref | ref |
| *Female* | 1.588 | 1.558-1.618*** | 1.748 | 1.722-1.774*** | 1.733 | 1.697-1.770*** | 1.711 | 1.665-1.758 | 1.949 | 1.868-2.033*** |
| PHE Centre | – | *** | – | *** | – | *** | – | *** | – | *** |
| Year | – | *** | – | *** | – | *** | – | *** | – | ns |
| **Interaction Effects** | | | | | | | | | | |
| Age:Gender | – | *** | – | ** | – | *** | – | *** | – | *** |
| Age:Deprivation | – | *** | – | *** | – | *** | – | *** | x | x |
| **R-Squared** | | | | | | | | | | |
| **Marginal** | | 0.864 | | 0.813 | | 0.303 | | 0.227 | | 0.993 |
| **Conditional** | | 0.941 | | 0.915 | | 0.959 | | 0.945 | | - |
| **Dispersion statistics** | | 1.1572 | | 1.262 | | 1.088 | | 1.025 | | 1.100 |

**2STROBE 2007 (v4) Statement—Checklist of items that should be included in reports of *cross-sectional studies***

| **Section/Topic** | **Item #** | **Recommendation** | **Reported on page #** |
|---|---|---|---|
| **Title and abstract** | 1 | (*a*) Indicate the study's design with a commonly used term in the title or the abstract | 3 |
| | | (*b*) Provide in the abstract an informative and balanced summary of what was done and what was found | 3 |
| **Introduction** | | | |
| Background/rationale | 2 | Explain the scientific background and rationale for the investigation being reported | 5/6 |
| Objectives | 3 | State specific objectives, including any prespecified hypotheses | 6/7 |
| **Methods** | | | |
| Study design | 4 | Present key elements of study design early in the paper | 8-10 |
| Setting | 5 | Describe the setting, locations, and relevant dates, including periods of recruitment, exposure, follow-up, and data collection | 8 |
| Participants | 6 | (*a*) Give the eligibility criteria, and the sources and methods of selection of participants | 8 |
| Variables | 7 | Clearly define all outcomes, exposures, predictors, potential confounders, and effect modifiers. Give diagnostic criteria, if applicable | 8-10 |
| Data sources/ measurement | 8* | For each variable of interest, give sources of data and details of methods of assessment (measurement). Describe comparability of assessment methods if there is more than one group | 8/9 |
| Bias | 9 | Describe any efforts to address potential sources of bias | *9/10* |
| Study size | 10 | Explain how the study size was arrived at | NA – surveillance system used which covers large proportion of population |
| Quantitative variables | 11 | Explain how quantitative variables were handled in the analyses. If applicable, describe which groupings were chosen and why | 10/11 |
| Statistical methods | 12 | (*a*) Describe all statistical methods, including those used to control for confounding | 10/11 |
| | | (*b*) Describe any methods used to examine subgroups and interactions | 10/11 |

For peer review only

| | | | |
|---|---|---|---|
| | | (*c*) Explain how missing data were addressed | 9/10 & Table 2 on page 20 |
| | | (*d*) If applicable, describe analytical methods taking account of sampling strategy | NA |
| | | (*e*) Describe any sensitivity analyses | 11 |
| **Results** | | | |
| Participants | 13* | (a) Report numbers of individuals at each stage of study—eg numbers potentially eligible, examined for eligibility, confirmed eligible, included in the study, completing follow-up, and analysed | 9 & 12 & Table 1 on page 19& Table 2 on page 20 |
| | | (b) Give reasons for non-participation at each stage | Table 2 on page 20 |
| | | (c) Consider use of a flow diagram | NA |
| Descriptive data | 14* | (a) Give characteristics of study participants (eg demographic, clinical, social) and information on exposures and potential confounders | 13/14 |
| | | (b) Indicate number of participants with missing data for each variable of interest | 12 & Table 2 on page 20 |
| Outcome data | 15* | Report numbers of outcome events or summary measures | 12 |
| Main results | 16 | (*a*) Give unadjusted estimates and, if applicable, confounder-adjusted estimates and their precision (eg, 95% confidence interval). Make clear which confounders were adjusted for and why they were included | 13/14 & Appendix 7 |
| | | (*b*) Report category boundaries when continuous variables were categorized | 13/14 |
| | | (*c*) If relevant, consider translating estimates of relative risk into absolute risk for a meaningful time period | NA |
| Other analyses | 17 | Report other analyses done—eg analyses of subgroups and interactions, and sensitivity analyses | 13/14 |
| **Discussion** | | | |
| Key results | 18 | Summarise key results with reference to study objectives | 15 |
| Limitations | 19 | Discuss limitations of the study, taking into account sources of potential bias or imprecision. Discuss both direction and magnitude of any potential bias | 17-18 |
| Interpretation | 20 | Give a cautious overall interpretation of results considering objectives, limitations, multiplicity of analyses, results from similar studies, and other relevant evidence | 15-17 |
| Generalisability | 21 | Discuss the generalisability (external validity) of the study results | 17 |
| **Other information** | | | |
| Funding | 22 | Give the source of funding and the role of the funders for the present study and, if applicable, for the original study on which the present article is based | 21 |

For peer review only

*Give information separately for cases and controls in case-control studies and, if applicable, for exposed and unexposed groups in cohort and cross-sectional studies.

**Note:** An Explanation and Elaboration article discusses each checklist item and gives methodological background and published examples of transparent reporting. The STROBE checklist is best used in conjunction with this article (freely available on the Web sites of PLoS Medicine at http://www.plosmedicine.org/, Annals of Internal Medicine at http://www.annals.org/, and Epidemiology at http://www.epidem.com/). Information on the STROBE Initiative is available at www.strobe-statement.org.

For peer review only
